# Recovery Following Recurrent Fires Across Mediterranean Ecosystems

**DOI:** 10.1111/gcb.70013

**Published:** 2024-12-27

**Authors:** Tiago Ermitão, Célia M. Gouveia, Ana Bastos, Ana C. Russo

**Affiliations:** ^1^ Faculdade de Ciências Instituto Dom Luiz, Universidade de Lisboa Lisbon Portugal; ^2^ Instituto Português do Mar e da Atmosfera, IPMA Lisbon Portugal; ^3^ Department of Biogeochemical Integration Max Planck Institute for Biogeochemistry Jena Germany; ^4^ Institute for Earth System Science and Remote Sensing, Leipzig University Leipzig Germany; ^5^ Associate Laboratory TERRA, CEF – Forest Research Centre School of Agriculture, University of Lisbon Lisboa Portugal

**Keywords:** climate variability, EVI, fire severity, post‐fire recovery, pre‐fire vegetation conditions, remote‐sensing

## Abstract

In fire‐prone regions such as the Mediterranean biome, fire seasons are becoming longer, and fires are becoming more frequent and severe. Post‐fire recovery dynamics is a key component of ecosystem resilience and stability. Even though Mediterranean ecosystems can tolerate high exposure to extreme temperatures and recover from fire, changes in climate conditions and fire intensity or frequency might contribute to loss of ecosystem resilience and increase the potential for irreversible changes in vegetation communities. In this study, we assess the recovery rates of burned vegetation after recurrent fires across Mediterranean regions globally, based on remotely sensed Enhanced Vegetation Index (EVI) data, a proxy for vegetation status, from 2001 to 2022. Recovery rates are quantified through a statistical model of EVI time‐series. This approach allows resolving recovery dynamics in time and space, overcoming the limitations of space‐for‐time approaches typically used to study recovery dynamics through remote sensing. We focus on pixels burning repeatedly over the study period and evaluate how fire severity, pre‐fire vegetation greenness, and post‐fire climate conditions modulate vegetation recovery rates of different vegetation types. We detect large contrasts between recovery rates, mostly explained by regional differences in vegetation type. Particularly, needle‐leaved forests tend to recover faster following the second event, contrasting with shrublands that tend to recover faster from the first event. Our results also show that fire severity can promote a faster recovery across forested ecosystems. An important modulating role of pre‐fire fuel conditions on fire severity is also detected, with pixels with higher EVI before the fire resulting in stronger relative greenness loss. In addition, post‐fire climate conditions, particularly air temperature and precipitation, were found to modulate recovery speed across all regions, highlighting how direct impacts of fire can compound with impacts from climate anomalies in time and likely destabilise ecosystems under changing climate conditions.

## Graphical abstract

Mediterranean ecosystems worldwide are frequently affected by fires. Focusing on areas that burned two times over the past 22 years, we study the post‐fire dynamics, applying a statistical model to the time‐series of a remotely sensed proxy for vegetation cover. Using this model, we assess and compare the recovery rates of different vegetation types, as well as determine the importance of fire severity and pre‐fire vegetation state on modulating the recovery rates.

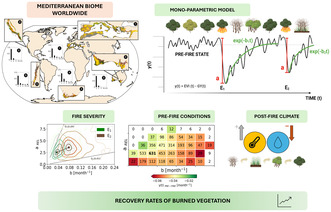

## 1 Introduction

Warmer and drier climate conditions in the past decades have been changing fire regimes (Pausas and Keeley 2021; Scholten et al. 2021; Jones et al. 2022, 2024), with extreme events associated with unprecedented fires in many ecosystems worldwide (Scholten et al. 2021; Byrne et al. 2024; Jones et al. 2024). These have been particularly evident in semi‐arid areas across the globe, such as the Mediterranean regions. For instance, in Europe, severe fire seasons in 2003, 2017 and 2022 occurred in Portugal and Spain (Gouveia et al. 2012; Turco et al. 2019; Ermitão et al. 2022, 2023; Ramos et al. 2023; Rodrigues et al. 2023), and in Greece and southern Italy during 2007, 2021, and 2023 (Gouveia et al. 2016; Evelpidou et al. 2021; Dosiou et al. 2024). Destructive wildfires also occurred in California in 2018, 2020 and 2021 (Brown et al. 2020; Higuera and Abatzoglou 2021), in Chile in 2017 and more recently in 2023 and 2024 (McWethy et al. 2018; Bowman et al. 2019; Cordero et al. 2024), in South Africa in 2021 (Liu et al. 2023) and in Australia, both in Mediterranean‐type ecosystems in 2015 (Etchells et al. 2020) and non‐Mediterranean‐type vegetation in 2006–2007 and 2019–2020 (McCarthy, Plucinski, and Gould 2012; Filkov et al. 2020).

Mediterranean ecosystems tend to experience frequent fires, with return intervals shorter than 20 years (Archibald et al. 2013; Harrison et al. 2021) and plants often exhibit fire adaptations such as thick barks, resprouting ability, or stimulation of reproduction after fire exposure (Lawes and Clarke 2011; Clarke et al. 2013; Pausas 2015; Harrison et al. 2021; Nolan et al. 2021). However, increasing frequency and severity of weather and climate extremes (Zscheischler et al. 2018; Vogel et al. 2021) can result in repeated impacts on ecosystems, so that their recovery might be limited (Davis, Shaw, and Etterson 2005; Brito‐Morales et al. 2018; Bastos et al. 2020, 2021). Compound effects of repeated weather extremes and changes in disturbance regimes can thus increase the potential for irreversible changes in ecological communities (Seidl and Turner 2022; Bastos et al. 2023). Therefore, the effects of recurrent extremes on ecosystem resilience have been gaining increasing attention (Anderegg, Kane, and Anderegg 2013; Falk, Watts, and Thode 2019; Steel et al. 2021; Bastos et al. 2021).

Ecological resilience can be decomposed into three different stages (Falk et al. 2022): *persistence*, the ability to tolerate exposure to the fire, *recovery*, which occurs when *persistence* is overcome and the vegetation must recover by reproduction e.g., recruitment, seed dispersal, and *reorganisation*, that occurs when *persistence* and *recovery* fail, and the ecosystem reorganises into a new state. While recovery dynamics is central to understanding ecosystem resilience, it is a complex process, being controlled by multiple factors such as disturbance impacts and climate (Meng et al. 2018; Bright et al. 2019; Nolan et al. 2021), but also land cover (Díaz‐Delgado et al. 2002; Epting and Verbyla 2005), topography (Wittenberg et al. 2007; Meng et al. 2015; Liu 2016) or the distance to seed banks (Donato et al. 2009). Moreover, human intervention and soil and land management after fire can modulate recovery dynamics (Baeza et al. 2007; Vieira et al. 2023).

Remote sensing has been widely used to study fire impacts and recovery dynamics (Díaz‐Delgado and Pons 2001; Díaz‐Delgado et al. 2002; Wittenberg et al. 2007; Gouveia, DaCamara, and Trigo 2010; Gouveia et al. 2012; Gouveia, Páscoa, and DaCamara 2018; Bastos et al. 2011; De Luca, Silva, and Modica 2022) as well as the influence of post‐fire climate conditions, topography or fire severity in some regions (Díaz‐Delgado et al. 2002; Liu 2016; Viana‐Soto, Aguado, and Martínez 2017). Regional studies have pinpointed the synergy between fire severity, pre‐fire vegetation state, and climate conditions on vegetation recovery: Bright et al. (2019) in western USA, Viana‐Soto et al. (2020) in Spain, and Rifai et al. (2024) in Australia. At a larger scale, Bousquet et al. (2022) analysed the recovery of different ecosystems worldwide after a fire event, by comparing different vegetation‐related variables such as Enhanced Vegetation Index (EVI), Leaf Area Index (LAI), and above‐ground biomass (AGB). Analysis of recovery dynamics at larger scales typically relies on space‐for‐time substitution approach, assuming the stationarity of recovery dynamics following fire events, and cannot resolve changes in recovery dynamics, e.g., due to varying climate conditions during the recovery periods, not temporally compounding effects of recurring fires (Moreno‐Mateos et al. 2020).

So far, no comprehensive assessment of recovery dynamics across space and time, including the effects of climate variability during the process has been performed for a broader region. Such information is especially relevant in regions recurrently disturbed by fires and exposed to climate change effects, such as the Mediterranean biome. Therefore, we provide a first comprehensive analysis of recovery dynamics of vegetation following recurrent fires over Mediterranean ecosystems worldwide based on a remotely sensed proxy for vegetation cover, the EVI over the period 2001–2022. With this approach, we also aim to evaluate how vegetation recovery is modulated by fire severity, pre‐fire state of vegetation, and post‐fire meteorological conditions. This framework intends to address the main scientific questions: (i) are the recovery processes of fire‐prone Mediterranean ecosystems being impacted by recurrent fires in recent years? (ii) how does the pre‐fire condition of these ecosystems influence fire severity, and in turn, how does fire severity modulate the recovery rate of burned vegetation? (iii) how can post‐fire meteorological conditions affect the recovery rate among the Mediterranean ecosystems worldwide?

## 2 Data

### 2.1 Biome

We use the definition of Mediterranean biome by Dinerstein et al. (2017), which is an updated version of the original biome map of 2001 by Olson et al. (2001). The dataset by Dinerstein et al. (2017) provides updated information of the 846 global ecoregions, nested within the 14 global biomes, where each one is defined based on homogeneous climate and vegetation characteristics.

### 2.2 Burned Area

To analyse the occurrence of fires over global Mediterranean ecosystems, we use monthly burned area and burn date uncertainty maps in each fire season from MODIS version 6.1, MCD64A1 (Giglio et al. 2018) between 2001 and 2022. The data have a spatial resolution of 500 m in its native projection and are generated using daily surface reflectance dynamics and thermal anomalies, allowing the detection of the most likely fire date. Several improvements to the algorithm have been integrated into the latest collection, especially regarding small fire detection and also the reduction of uncertainty on burn‐date guessing.

Data used here were extracted for the seasonal extended fire seasons, according to information provided in the Global Wildfire Information System (GWIS) Portal (https://gwis.jrc.ec.europa.eu/apps/country.profile/, accessed on 30 June 2023) in each of the five selected regions: in the Mediterranean basin and California, the typical fire seasons extend from June to October, in Australia between October and February while in South Africa and Chile extend from December to April.

### 2.3 Vegetation Indices

EVI quantifies the greenness of the land surface and provides a proxy for vegetation cover and condition, including several adjustments regarding the canopy background and atmospheric corrections and showing lower saturation over high‐density canopies compared to other vegetation spectral indices (Huete et al. 2002; Didan et al. 2015; Xu et al. 2020). Therefore, EVI is a suitable index to assess the recovery of vegetation after disturbances due to its strong sensitivity to seasonal or biophysical changes (Wittenberg et al. 2007; Veraverbeke et al. 2012; Pérez‐Cabello, Montorio, and Alves 2021).

Here, we use EVI from MODIS Collection 6.1 (MOD13A1, Didan et al. (2015)) to monitor vegetation dynamics, fire severity, and recovery. MODIS EVI is provided as 16‐day composites at a spatial resolution of 500 m in its native projection. For our study, we extracted the EVI between 2001 and 2022, to match the period available for burned areas.

### 2.4 Land Cover

The land‐cover distribution analysis over the Mediterranean biome is based on the land‐cover product from ESA CCI Land Cover, CCI‐LC (Kirches et al. 2014), which provides global land cover at a spatial resolution of 300 m and is available between 1992 and 2020. By combining global surface reflectance data from five different Earth Observation systems (Liu et al. 2018), the product describes the surface cover in 37 original LC classes following the United Nations Land Cover Classification System (UN‐LCCS, Di Gregorio 2005; Liu et al. 2018). For our work, the annual maps for the period 2001–2020 were extracted. Considering that CCI‐LC is available up to 2020, we used the 2020 land‐cover classification map for 2021 and 2022.

### 2.5 Climate Data

We use precipitation and 2 m air temperature data from the ERA5‐Land reanalysis at 9 km (0.10°) spatial resolution, available since 1950 (Muñoz‐Sabater et al. 2021). ERA5‐Land is a downscale product based on ERA5 and shows strong performance on energy and hydrological cycle modulation, with higher spatial resolution and longer temporal coverage (Muñoz‐Sabater et al. 2021), allowing us to better characterise the post‐fire meteorological conditions in our study. The meteorological variables, such as precipitation and 2 m air temperature from ERA5‐Land, have been recently used to characterise the recovery of burned vegetation, for example, in Spain (Rodrigues et al. 2023) or in Australia (Rifai et al. 2024). In our study, we selected the period 2001–2022 to match the period selected for vegetation and burned areas data.

## 3 Methods

### 3.1 Study Region

**FIGURE 1:**
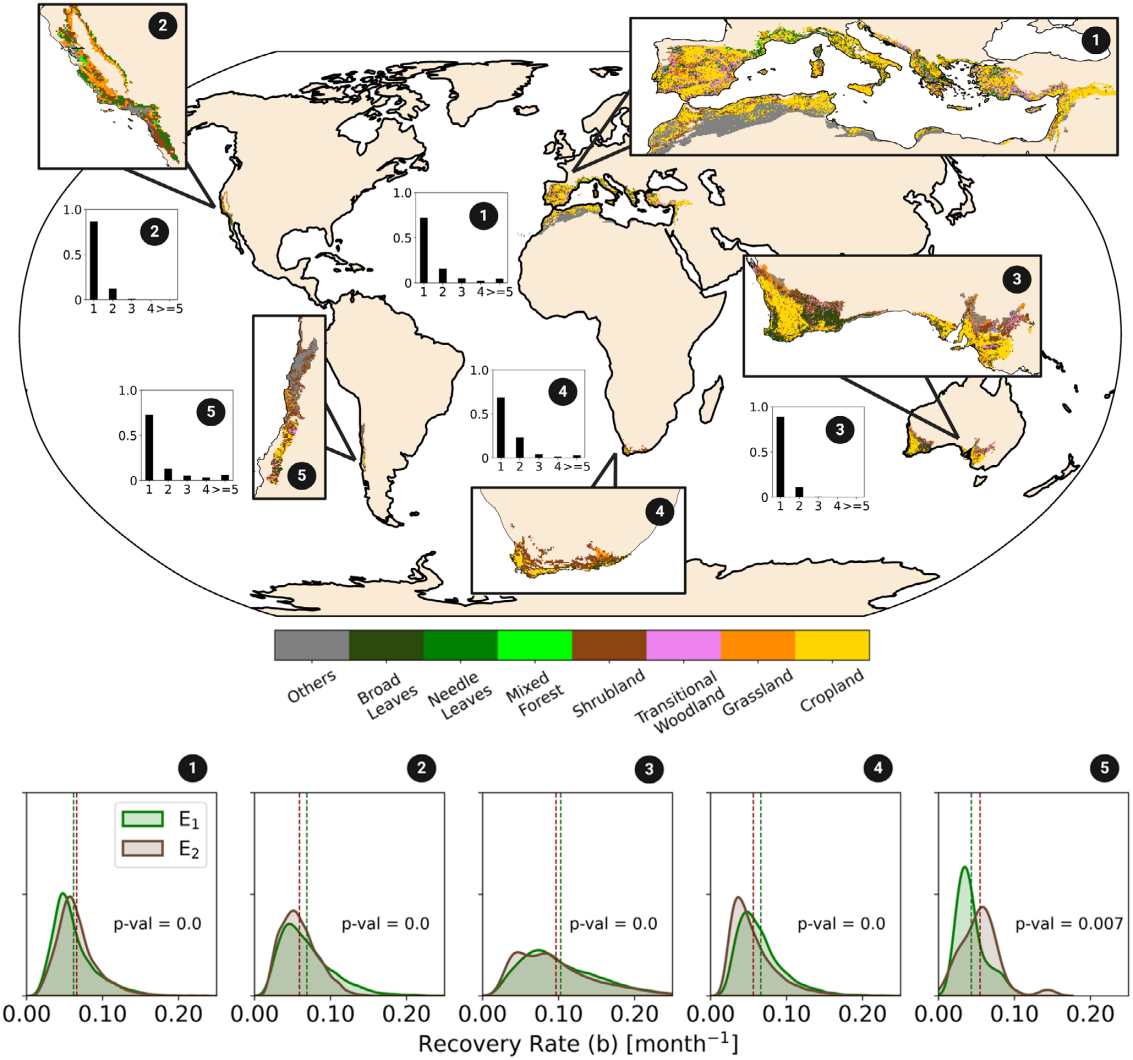
Top panel: Global distribution of the defined categories of land cover over considering each region of the Mediterranean biome: 1—Mediterranean basin; 2—California; 3—Australia; 4—South Africa; 5—Chile. The relative fire frequency is represented on black bars for each region; Bottom panel: Probability Density Functions (PDFs) of recovery rate distribution of burned vegetation (parameter *b*, in month^−1^) for different regions following the first fire event, E_1_ (green curve) and second fire event, E_2_ (brown curve). In the *y*‐axis, the density of probability is represented. The p‐value of the Wilcoxon signed‐rank test to determine if the difference between distributions is statistically significant is represented for each region. Dashed vertical lines represent the mean recovery rate, *b*, for E_1_ (green) and E_2_ (brown). Map lines delineate study areas and do not necessarily depict accepted national boundaries.

We analysed fire recurrence over five Mediterranean regions across the globe: the Mediterranean basin, southern California, southwestern and southern Australia, southwestern South Africa, and along the coast of Chile (Figure 1, top panel). These regions usually have wet and mild to cool autumn and winter seasons, whereas the summers are characterised by hot and dry conditions (Figure S1), being essentially classified as *Csa* and *Csb* in the Köppen‐Geiger classification (Köppen 1936). The land‐cover distribution in these regions is heterogeneous (see Figure 1 for land‐cover distribution in regions within the Mediterranean biome). Needleleaf trees (*NL*) are predominant in California and the Mediterranean basin, especially in the Iberian Peninsula, while broad‐leaved trees (*BL*) are present in all regions except California, predominating in several areas of western Australia and parts of northern Africa near the coast. In turn, transitional woodland vegetation (*TW*) is mainly present in the Mediterranean basin, while shrublands (*Shb*) cover all regions, especially in South Africa or California.

### 3.2 Data Pre‐Processing

For the 16‐day EVI composites, the pre‐processing of data followed a technique that was previously proposed by Los (1998) and Stöckli and Vidale (2004) and was successfully applied to correct the NDVI time‐series from SPOT/VEGETATION by Gouveia, Trigo, and DaCamara (2009); Gouveia, DaCamara, and Trigo (2010). Low‐quality pixels (contaminated by clouds, snow, ice, or other issues) were primarily replaced through spatial interpolation, and then the EVI time‐series in each pixel of the composite was adjusted by using a temporal cubic interpolation procedure. Despite the quality correction, strong fluctuations in time‐series at pixel‐scale were still found. Therefore, an unweighted second‐order Fast Fourier Transformation (FFT) was applied, aiming to remove the noise but retaining the small‐scale variations, as well as the signature of disturbances in vegetation. Vegetation indexes show long‐term trends, which can be due to different drivers (Zhu et al. 2016), and such long‐term trends would influence the estimate of recovery times, especially when comparing recurrent events within a time‐series with 20 years long. Therefore, aiming to reduce these effects, we detrended each pixel's time‐series using a locally estimated scatterplot smoothing technique (LOESS technique, Cleveland et al. 1990).

Vegetation indices and climate data were aggregated into 16‐day composites, re‐gridded to geographical projection, and resampled into 0.005° latitude × 0.005° longitude resolution. The annual land‐cover maps were resampled to the same spatial resolution using majority resampling. We aggregated the original 37 classes of land cover from CCI‐LC into seven main categories, according to different characteristics of vegetation, namely: Broad‐leaved, *BL* (classes 50, 60); Needle‐leaved, *NL* (classes 70, 80); Mixed Forest, *MF* (class 90); Shrubland, *Shb* (classes 40, 110, 120); Transitional Woodland, *TW* (class 100); Grassland, *Gs* (class 130); Cropland, *Cp* (classes 10, 20, 30); and Others, *Oth* (classes between 150 and 220).

### 3.3 Fire Recurrence

Following the identification of the global areas classified as Mediterranean biome, we then monitored the burned areas within those ecosystems. The MCD64A1 dataset has inherent uncertainty from optical surface reflectance measurements and thermal anomalies (Brennan et al. 2019) that can introduce additional inaccuracy in burned areas detection (Vermote, El Saleous, and Justice 2002). Thus, after a sensitivity analysis, burned pixels with an uncertainty date of burn detection longer than 7 days were excluded.

First, we quantified fire frequency between 2001 and 2022 across the whole Mediterranean biome (see Figure 1). We are interested in assessing potential changes in recovery rates over the study period. Therefore, we focus on pixels burning more than once. At the same time, the time‐series is still relatively short (22 years) compared to the typical recovery periods of certain vegetation types across Mediterranean ecosystems (about 20 years, Archibald et al. 2013). For instance, Gouveia, DaCamara, and Trigo (2010); Gouveia, Páscoa, and DaCamara (2018) and Bastos et al. (2011) estimated that the NDVI of several burned areas following the fire events of 2003 and 2005 in Portugal recovered on average to pre‐fire greenness levels in 3–5 years (though some areas took longer). Similarly, Viana‐Soto, Aguado, and Martínez (2017) and Hislop et al. (2018) estimated 5–7 years for forests in the Iberian Peninsula to recover to pre‐fire greenness levels. Lastly, Bousquet et al. (2022) reported that Australian eucalyptus took approximately 3 years to recover to pre‐fire EVI levels. Therefore, as our statistical approach requires a long‐enough period post‐fire to estimate recovery time robustly (see sections below), we focus on pixels that burned twice over the study period, and occurred at least four years apart.

### 3.4 Recovery Model

**FIGURE 2:**
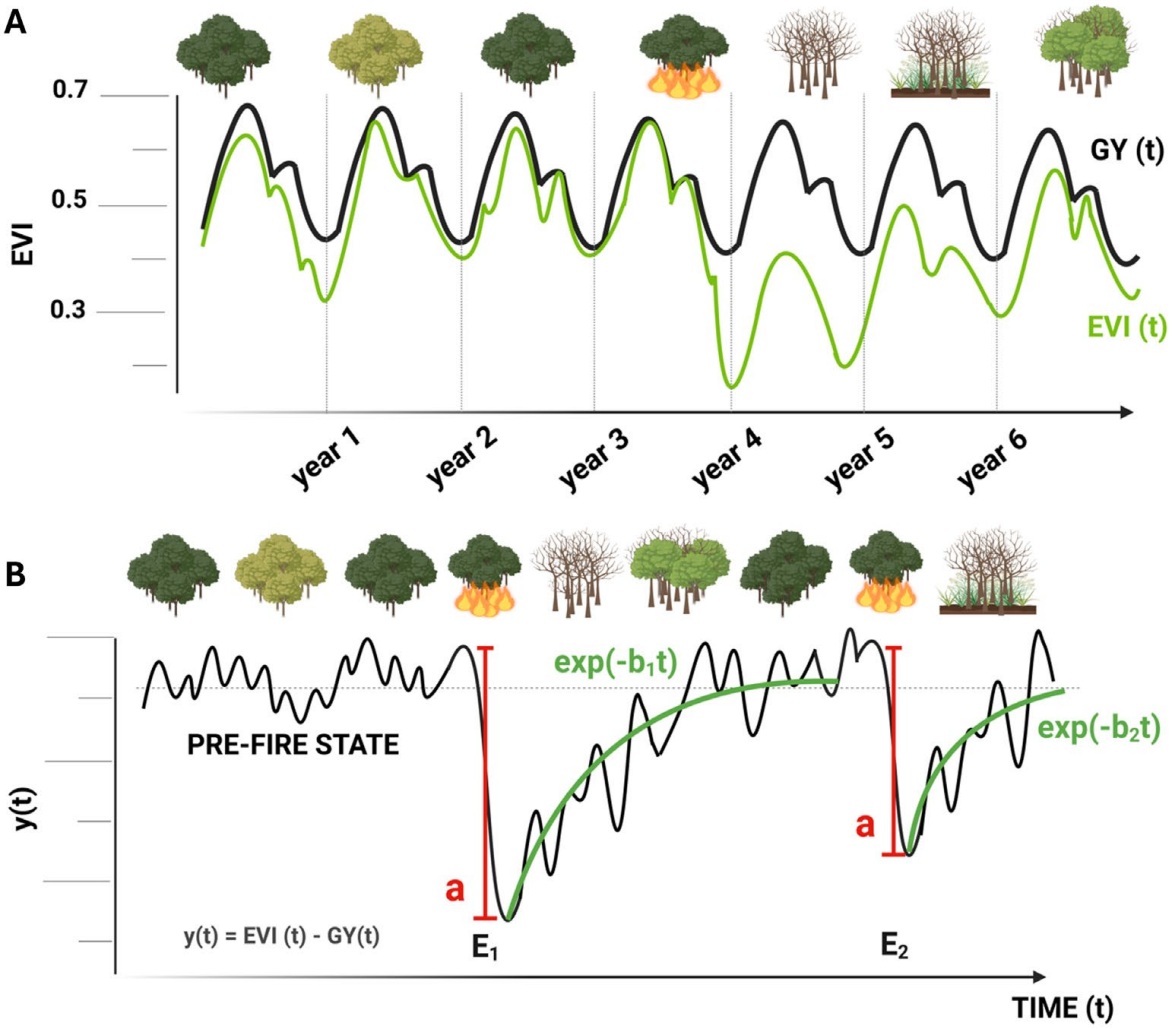
(A) Schematic representation of the time‐series of monthly EVI (green curve) and monthly GY (black curve) and the impact of a fire event in one pixel. (B) Schematic representation of the impact of two fire events in the time‐series of loss of greenness, *y*(*t*). The largest loss of greenness caused by fire is represented by a (red vertical lines) for the first fire event (E_1_) and second fire event (E_2_). The adjusted recovery model for each event is represented in green curves, and the model's equation is also described. The mean pre‐fire state of the ecosystem is characterised by the black dashed line. Figure created with BioRender.com.

We followed the approach proposed by Gouveia, DaCamara, and Trigo (2010) to estimate post‐fire recovery based on a statistical regression model of vegetation greenness following fire. First, we aggregated the 16‐day composites of EVI per month and calculated, at the pixel‐level, the departure of observed EVI to an ideal seasonal cycle, referred to as the Gorgeous Year, GY(*t*) (see Figure 2 for schematic representation). This annual vegetative cycle GY(*t*) is defined by estimating the highest EVI value in each monthly interval over the period 2001–2022 (Figure 2A). The normalisation of observed EVI by the GY(*t*) over time generates a time‐series, hereafter named as *y*(*t*), and defined as:

(1) $$y\left(t\right)=\mathrm{EVI}\left(t\right)-\mathrm{GY}\left(t\right)$$

Therefore, *y*(0) ranges between −1 and 0, and, the further it departs from zero, the greater the loss of greenness at a given pixel, relative to its ideal state.

To estimate post‐fire recovery, it is assumed that the greenness recovery rate, dy/dt, is proportional to the loss of greenness *y*(*t*):

(2) $$\frac{\mathrm{dy}}{\mathrm{dt}}=-\mathrm{by}$$

Or, integrating:

(3) $$y\left(t\right)=ae^{-\mathrm{bt}}$$

The parameter *a* corresponds to the minimum of *y*(*t*), i.e., the maximum loss of greenness associated with fire, whereas the parameter *b* expresses the recovery rate following the fire event (Figure 2B). A “characteristic recovery time” can also be derived by inverting the mean recovery rate (1/*b*), corresponding to the time in which ca. 50% of relative recovery is reached.

It is worth noting that the recovery rates estimated here correspond to the recovery of surface greenness as seen by optical remote‐sensing, not necessarily the full recovery in terms of accumulated biomass and ecosystem functioning. These are expected to take longer (Bousquet et al. 2022; Fan et al. 2023).

### 3.5 Model Fitting

Vegetation indexes can monitor the impact of fire events on ecosystems (Gouveia, DaCamara, and Trigo 2010; Gouveia et al. 2012; Gouveia, Páscoa, and DaCamara 2018; Bastos et al. 2011; Bright et al. 2019). However, sharp anomalies in time‐series that might result from factors such as satellite‐sensor drifts, human interventions, land‐cover changes, or other climatic extreme events, were detected. To ensure that the loss of greenness corresponds to the impact of the fire, we estimated the minimum value of *y*(*t*) between the fire occurrence and the immediate weeks after the fire event.

The recovery rate *b* of each pixel can be estimated by means of the slope of the linear regression applied to the ln(*y*(*t*)) in the years following the occurrence of *a*:

(4) $$\ln\left(\frac{y\left(t\right)}{a}\right)=-\mathrm{bt}$$

The parameter *b* is estimated iteratively, and based on the linear regression model showing the best performance (given by the adjusted *r* ^2^), when fitting to periods of different lengths (2–5 years) following the occurrence of the minimum of *y*(*t*). This iterative approach implies that different post‐fire period lengths are selected for each pixel, depending on the variability of *y*(*t*), but ensure that the most robust model is used. For some events, the statistical model showed poor fit to *y*(*t*), hindering robust quantification of recovery rates, therefore, we excluded pixels in which both statistical model's event showed adjusted *r* ^2^ values lower than 0.25.

We analysed the results separately for the following land‐cover categories: *BL*, *NL*, *Shb*, and *TW*, based on the per‐pixel classification in the year before the fire event. We assumed that the land‐cover category of the pixel is the same for the two events, disregarding any land‐cover shifts that may happen between fires.

The selected number of points for the recovery model of each land‐cover category and each region of the domain are described in Table 1.

**TABLE 1:** Number of pixels in each land‐cover category in each region of the domain. The total number of points for each category is also represented.

	BL	NL	Shb	TW
Mediterranean	2730	5040	2580	7321
California	—	4330	3380	11
Australia	10,349	—	2098	775
South Africa	271	—	5345	151
Chile	21	—	32	6
Total	13,371	9370	13,435	8264

The performance of the statistical model for E_1_ and E_2_, evaluated using squared regression coefficient, *r* ^2^, for each land‐cover category, as well as for each fire event, is described in Figure S2. The model exhibits, for both events, a higher median performance for *NL* (*r* ^2^ of about 0.65), although with slight differences between fire events, followed by *BL* and *TW*. In *Shb*, the performance is lower, showing a median *r* ^2^ of about 0.50. Across the four land‐cover categories, the r^2^ distribution generally spans from 0.30 to 0.80, exhibiting some event‐specific variations. The model skill also shows regional differences (map of Figure S3), with the Mediterranean basin, particularly in Portugal and northern Africa, and California showing the best model fit and some areas in South Africa showing lower values of the *r* ^2^, indicating a poorer model adjustment to the data.

The confidence intervals (CI) of recovery rates with a statistical significance of 5% are also provided in Figure S4. Across all the categories, CI increases with the recovery rate increase, indicating higher uncertainty of recovery rate estimation for exceptionally fast recovery rates. Some evident differences among the land‐cover categories are found, particularly in *BL*, which shows larger uncertainties over a range of points that have very fast recovery rates. On the contrary, almost all points of *NL* have low CI, ranging mainly between 0 and 0.03 month^−1^, showing the goodness‐of‐fit in this case.

Alongside r‐square information, an overall good performance of the statistical model applied to the 5 regions of the domain is estimated.

### 3.6 Pre‐ and Post‐Fire Assessment

Post‐fire vegetation recovery can be modulated by a wide range of factors, namely fire severity, pre‐fire vegetation state, and post‐fire meteorological conditions (Bright et al. 2019; Viana‐Soto et al. 2020; Rifai et al. 2024).

After estimating recovery rates of burned vegetation (*b*), we calculated the differences between the recovery rate of the second (E_2_) and first event (E_1_) per‐pixel, $b_{\text{DIFF}}=b_{2}-b_{1}$. The nonparametric Wilcoxon signed‐rank test was applied to compare both distributions and to test if the differences are statistically significantly different from zero. Positive differences (*b*_DIFF_ > 0) indicate faster recovery after E_2_ than E_1_ (hereafter *b*_1_ < *b*_2_), while negative differences (*b*_DIFF_ < 0) indicate faster recovery following E_1_ than E_2_ (hereafter *b*_1_ > *b*_2_). Hence, two groups of pixels were defined based on their recovery rate differences: those with *b*_1_ < *b*_2_ and those with *b*_1_ > *b*_2_. We analysed these two groups of pixels, *b*_1_ < *b*_2_ and *b*_1_ > *b*_2_, separately for each land cover‐type, selecting pixels with *b*_DIFF_ falling outside the 25th–75th interquartile range of the distribution to ensure that we sampled pixels where differences were most relevant. Employing more strict constraints, such as selecting differences outside the 10th–90th percentiles would produce similar results but with a smaller and less representative sample size. Therefore, further analysis of fire severity, pre‐fire conditions, and post‐fire climate variability was performed using the 25th–75th percentiles criteria.

The parameter *a* of the model fit is used as a proxy of fire severity. To compare across pixels with different greenness levels, we estimated the relative fire severity, *a*_REL_ as:

(5) $$a_{\text{REL}}=\left|\frac{a}{{\mathrm{GY}}_{\text{MEAN}}}\right|$$

where GY_MEAN_ is the mean of the annual seasonal cycle of GY at the pixel‐level. Due to the negative signal of absolute severity, *a*, we guarantee, by using the module, that the *a*_REL_ value ranges from 0 (no severity) towards positive infinity.

First, we analysed the relationship between fire severity and recovery rates by fitting a Kernel Density Estimator (KDE) to the bivariate distribution of *a*_REL_ and *b*, grouping pixels in each land‐cover class. We separate the two events to evaluate potential changes in this relationship over time and further assess the dependence of recovery rates of burned vegetation on fire severity by performing a quantile regression on the 1st (the least severe fires) and 99th (the most severe fires) quantiles of the relative fire severity distribution.

To analyse the influence of vegetation state before the fire on recovery rates, we estimated at pixel‐level the median of *y*(*t*) over the 3 months before the fire occurrence, *y*(*t*)_PRE‐FIRE_. This value roughly corresponds to the fuel accumulated before the fire season.

We further assess the relationship between recovery rates and post‐fire climate variability by analysing the evolution of seasonal standardised anomalies of precipitation and temperature over the 12 months following fire for each pixel and event. The seasonal standardised anomaly was calculated by removing the climatological seasonal mean 2001–2022 and dividing by the seasonal standard deviation 2001–2022. This analysis was conducted by calculating the anomalies at pixel‐level, considering each hemisphere's season. The anomalies were further grouped for each condition, i.e., *b*_1_ < *b*_2_ and *b*_1_ > *b*_2_ across the four land‐cover categories.

## 4 Results

### 4.1 Recovery Rate Assessment

Over the period 2001–2022, several Mediterranean areas experienced recurrent fires (Figure 1, top panel, bar plots): 20%–25% (more than 10%) of the total burned areas in the Mediterranean basin and South Africa (California, Chile, and Australia) burned twice (see also Figure S5). About 10% of the total burned area in this period in the Mediterranean basin, South Africa, and Chile burned three times or more. Frequent fires (more than once) are particularly relevant in the western regions of the Mediterranean basin (Portugal and northern Spain), as well as across southern California, southwestern Australia, and extensive areas of South Africa (Figure S5).

We find important differences in mean recovery rates between the selected regions (Figure 1, bottom panels), with Australia showing the fastest recovery rates (on average 0.10 month^−1^ and characteristic recovery time of about 10 months) and Chile showing the slowest average recovery rates (about 0.04 month^−1^ and characteristic recovery time of 25 months). In the Mediterranean basin, California, and South Africa, the average recovery rate ranges from about 0.05 to 0.065 month^−1^, with a characteristic recovery time of about 15–20 months. Therefore, characteristic recovery times range between 1 year in Australia, 1 year and half in the Mediterranean basin, California, and South Africa, and 2 years in Chile. In the Mediterranean basin, South Africa, and also in California, recovery rates show narrower distributions, indicating more similar recovery rates across pixels within each region, whereas in Australia the flatter distribution indicates larger regional variability in recovery rates (Figure 1, bottom panel).

To evaluate whether there are temporal changes in recovery dynamics, we also compare the distribution of the recovery rate of burned vegetation following each individual fire event in pixels burned twice (Figure 1, bottom panel). On average, recovery tends to be faster following the first event (E_1_) compared to the second (E_2_) in most regions, except for Chile and the Mediterranean basin, where similar mean recovery rates between the two events are found. Spatially, pixels with larger differences in recovery rates are found particularly in the western sector of the european Mediterranean basin (Portugal and northern Spain), where vegetation recovered faster after E_2_ than E_1_, and in southern California and western South Africa, where vegetation shows slower recovery after E_2_ than E_1_ (Figures S6 and S7).

**FIGURE 3:**
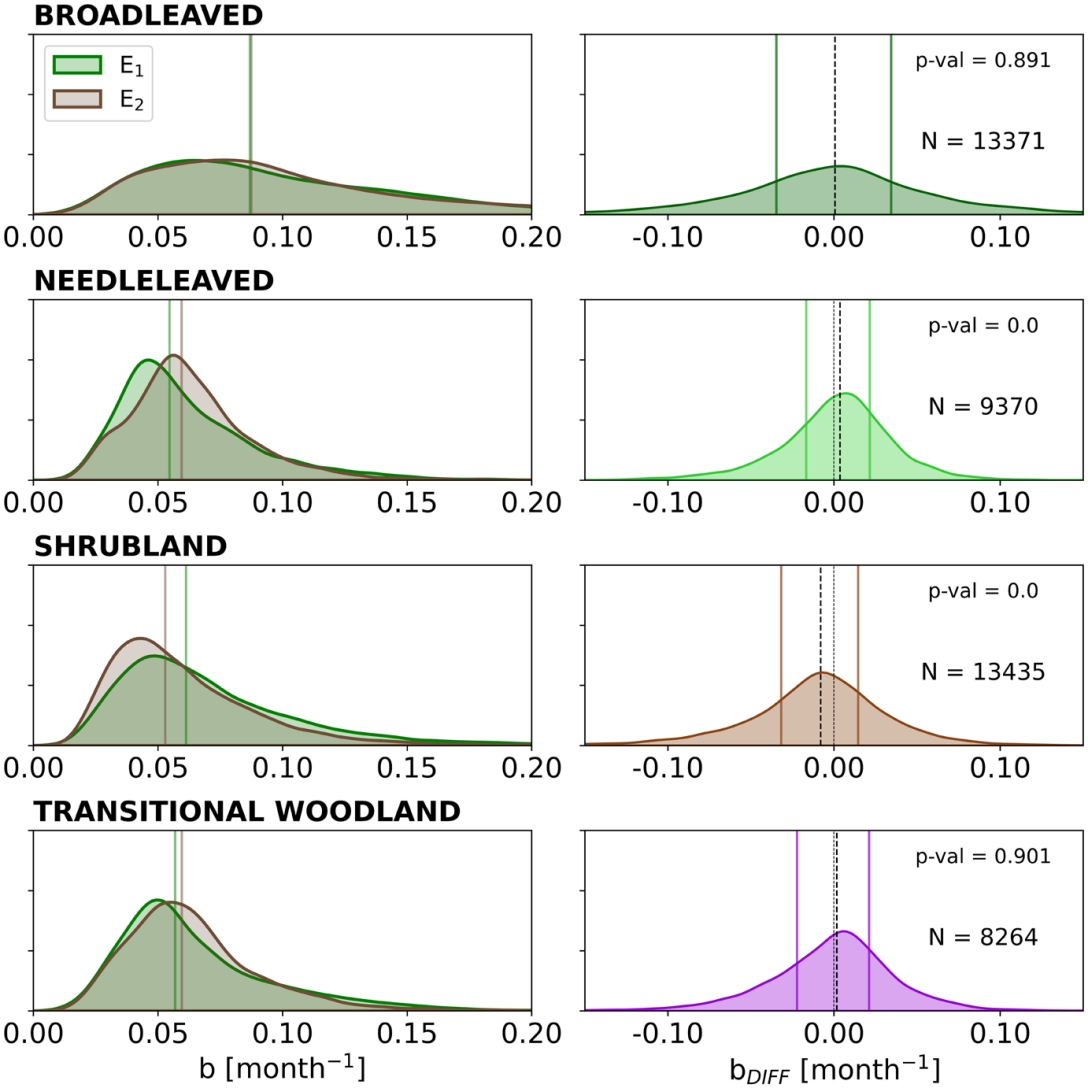
Left panels: Distribution of the recovery rates (*b*, month^−1^) of vegetation considering the selected land cover categories for the first fire event, E_1_ (green curve), and the second event, E_2_ (brown curve). The vertical lines represent the median of each distribution; Right panels: Distribution of differences in recovery rates between the two fire events, *b*_DIFF_. The black dashed line describes the median value of *b*_DIFF_ and the thin grey dashed line is the vertical line of *b*_DIFF_ = 0. The bold lines correspond to the 25th (left side of the distribution) and 75th (right side of the distribution) percentiles of *b*_DIFF_ in each category. The p‐value corresponds to the Wilcoxon signed‐rank test applied with a 95% significance.

We then compare the dependence of recovery rates on land cover (Figure 3). We find that Needle‐leaved forests (*NL*), Shrubland (*Shb*), and Transitional Woodlands (*TW*) tend to show recovery rates with mean values ranging between about 0.05 and 0.065 month^−1^, corresponding to characteristic recovery times of ca. 15–20 months. In turn, Broad‐leaved forests (*BL*) tend to show faster recovery times (0.08 month^−1^ and characteristic recovery times of 12 months), as well as larger variability in recovery rates than the other vegetation types, as seen by the broader distributions of recovery rates.

For *NL* and *Shb*, the distributions of the recovery rates also reveal important differences between fire events. For *NL*, 50% of the pixels show recovery rates faster than 0.059 month^−1^ for E_2_, while for E_1_ this value is 0.054 month^−1^ (Figure 3, left panels). On average, *NL* generally show faster recovery following E_2_ than E_1_ by 0.005 month^−1^, as given by the mean value of the pairwise differences, but also as shown on the displacement of the median values for E_2_ compared to E_1_, and the left‐skewed distribution with positive median of paired differences (Figure 3, right panels). By contrast, in *Shb*, 50% of pixels are showing recovery rates on average faster than 0.053 month^−1^ for E_2_ while for E_1_, 50% of the distribution recovers at 0.061 month^−1^ or faster (Figure 3, left panel). Hence, *Shb* pixels recover faster after E_1_ than E_2_ by 0.008 month^−1^, as given by the mean value of the pairwise differences between and also as shown on the right‐skewed distribution and the negative median value of paired differences, which reflects more slower‐recovering pixels after E_2_ (Figure 3, right panels).

In *BL* and *TW*, the Wilcoxon signed‐rank test shows that the median differences between recovery rate distributions are not statistically significant, therefore indicating that E_1_ and E_2_ distributions are very similar. However, the broad spread of b_DIFF_ distributions can indicate that marked differences in recovery rates between the two events have occurred, but these cancel each other when aggregating spatially. Specifically, *BL* pixels with differences different from zero are found in the Mediterranean basin (positive median, left skewed distribution) and South Africa (negative median, right‐skewed distribution) and *TW* pixels with differences different from zero are found in the Mediterranean basin (positive median, left skewed distribution) and Australia (negative median, right‐skewed distribution) (Figure S8).

### 4.2 Fire Severity

**FIGURE 4:**
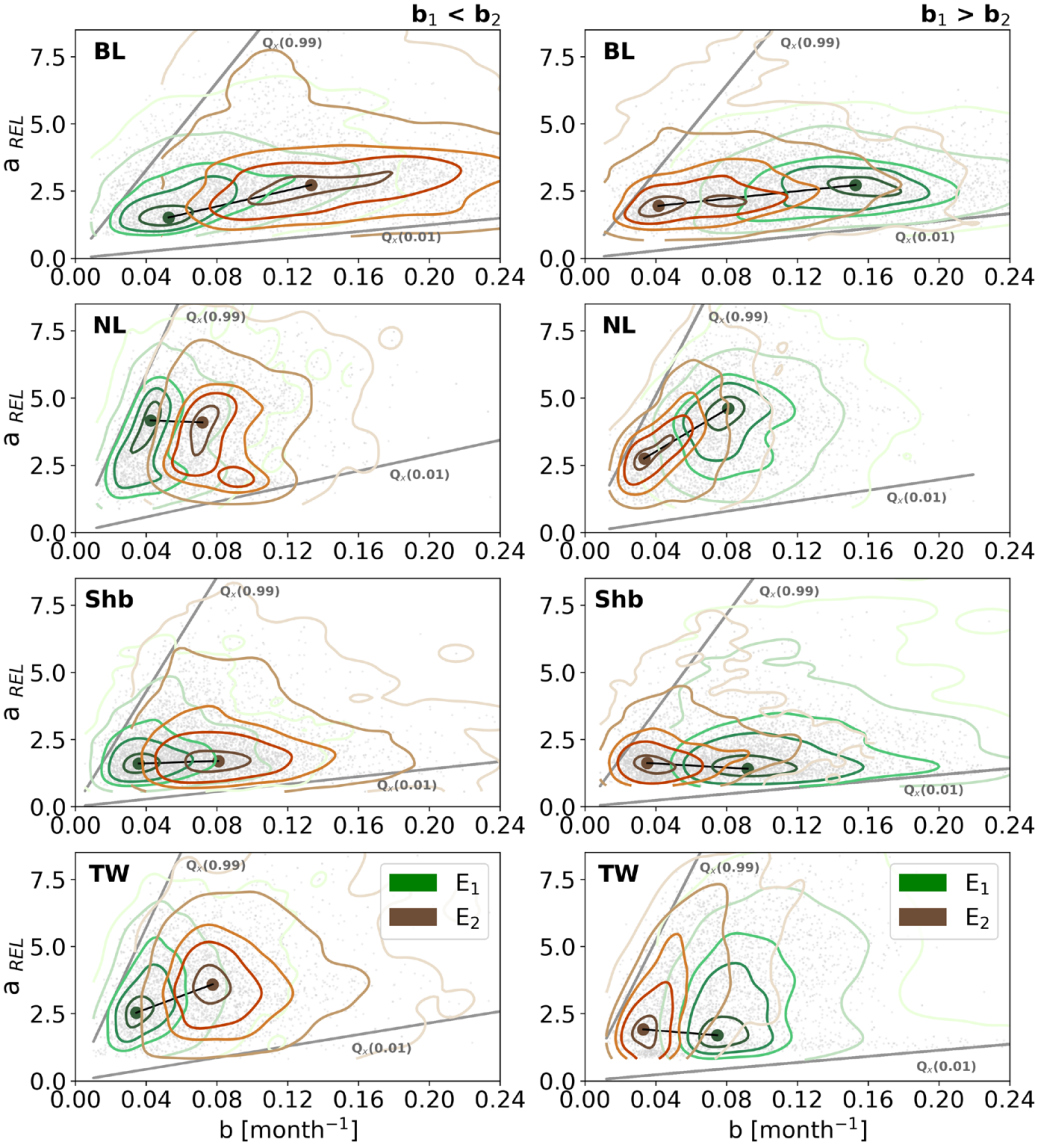
Kernel Density Estimates (KDEs) of the global bivariate distributions of greenness recovery rate, *b*, and relative severity, *a*_REL_, in each land‐cover category for E_1_ (green) and E_2_ (orange) for the group *b*_1_ < *b*_2_ (left‐side plots) and *b*_1_ > *b*_2_ (right‐side plots). The distribution of points of the sample of both events is described in grey points. The black line links the centroids of E_1_'s and E_2_'s KDEs. The grey lines correspond to the 0.01, Qx(0.01), and the 0.99, Qx(0.99) quantiles of the regression.

We find a general positive relationship between relative fire severity (*a*_REL_) and post‐fire recovery rate (*b*) for each condition and land cover‐type (grey points in Figure 4). However, the distribution of the points suggests a marked dependence of this relationship on the relative fire severity. Results of the quantile regression show a strong sensitivity of recovery rates to relative severity in the most severe fires (99th quantile of *a*_REL_) for all vegetation types, with slopes ranging between 81 and 147 month^−1^ per relative severity unit. In contrast, for the least severe fires (first quantile of *a*_REL_), the recovery rates show low sensitivity to relative severity, with slopes ranging between 6 and 14 month^−1^ (per relative severity unit). Although between the pixel's conditions *b*_1_ < *b*_2_ and *b*_1_ > *b*_2_ the differences are almost negligible, among the different land cover‐types, sharp contrasts within slopes are noticeable. The highest slopes of the quantiles regressions can explain the increasing dependence of the recovery rate of burned vegetation on fire severity. Specifically, *NL* shows the highest slope of the 99th quantile regression (147 and 127 month^−1^ per relative severity unit for *b*_1_ < *b*_2_ and *b*_1_ > *b*_2_, respectively), followed by *TW* (142 and 130 month^−1^ per relative severity unit for *b*_1_ < *b*_2_ and *b*_1_ > *b*_2_, respectively). Lower slopes of the 99th quantile regression are found in *Shb* (107 and 89 month^−1^ per relative severity unit for *b*_1_ < *b*_2_ and *b*_1_ > *b*_2_, respectively) and in *BL*, that shows the lowest values compared to the other land cover‐types (82 and 80 month^−1^ per relative severity unit for *b*_1_ < *b*_2_ and *b*_1_ > *b*_2_, respectively). The same is found for the first quantile regression, with *NL* slopes varying between 10 and 14 month^−1^ per relative severity unit considering both pixel's conditions, and *BL* ranging between 6 and 7 month^−1^ per relative severity unit.

Given the similar configuration of the bivariate relationship between fire severity and recovery rate (*a*_REL_, *b*) for different vegetation types and pixel's conditions, variations in recovery rates per land cover‐type and between consecutive events can be partly explained by differences in fire severity. Specifically, in *BL*, *NL* (*b*_1_ > *b*_2_) and *TW* (*b*_1_ < *b*_2_), the faster recovery rates following E_2_ than E_1_ are associated with a shift of the bivariate distribution towards higher severity. For the other vegetation types and pixel's conditions, the mean severity is comparable for the two events and, in the case of *Shb* and *TW* (*b*_1_ < *b*_2_) relatively small, so that the centroid of the bivariate distribution follows a displacement consistent with the slope of the lowest severity pixels. Exceptions are found in *NL* (*b*_1_ < *b*_2_), *Shb* and *TW* (*b*_1_ > *b*_2_), where the centroids of the bivariate distribution shifted almost horizontally, indicating that on average, relative severity is similar in both events, but other factors influence vegetation recovery rates.

### 4.3 Pre‐Fire Vegetation State

**FIGURE 5:**
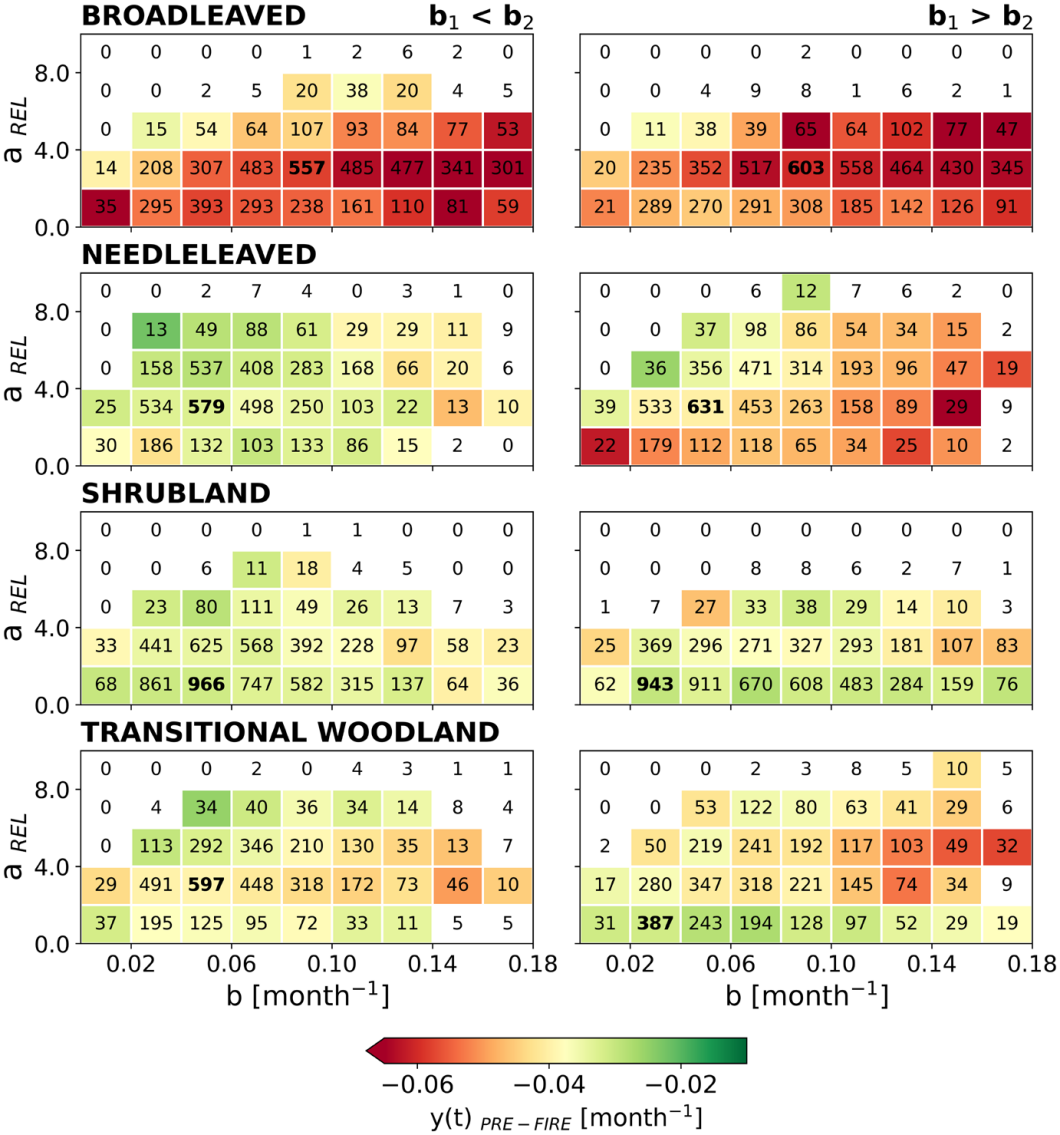
Diagram of the bivariate distribution of greenness recovery rate, *b*, and relative severity, *a*_REL_, binned for different levels of *y*(*t*)_PRE‐FIRE_, considering each land‐cover category when *b*_1_ < *b*_2_ in (left panels) and *b*_1_ > *b*_2_ (right panels). The number of points within each bin is described and the number represented in bold corresponds to the bin which presents the highest number of points.

We then analyse how pre‐fire vegetation condition, *y*(*t*)_PRE‐FIRE_, varies across different values of recovery rates and fire severity for each land‐cover category (Figure 5) and for the conditions *b*_1_ < *b*_2_ (left panel) and *b*_1_ > *b*_2_ (right panel).

Compared to other vegetation types, *BL* exhibits overall lower *y*(*t*)_PRE‐FIRE_, for both conditions across different levels of relative fire severity. In turn, *Shb* consistently shows a state closer to the ideal in both conditions and for most levels of *a*_REL_ and *b*, suggesting a weaker influence of pre‐fire conditions of vegetation on relative fire severity and thereby on recovery rate.

Predominantly larger departures from the ideal seasonal cycle are found for intermediate levels of relative severity in most vegetation types, which can be associated with higher availability of fine and dry fuels from leaf shedding under stress resulting in more intense fires. For similar levels of relative fire severity, more strongly negative values of *y*(*t*)_PRE‐FIRE_ tend to be associated with faster recovery rates for *BL* in both cases, for *NL* (*b*_1_ < *b*_2_) and for *Shb* (*b*_1_ > *b*_2_) and *TW* (*b*_1_ > *b*_2_), which can explain why the bivariate distribution of (*a*_REL_, *b*) in these cases shifts horizontally. On the other hand, closer to ideal seasonal cycle values of *y*(*t*)_PRE‐FIRE_ tend to be associated with more severe fires and slower recovery rates. This is observed for *NL* and *TW* in both conditions and *BL* (*b*_1_ < *b*_2_).

### 4.4 Post‐Fire Climate Variability

**FIGURE 6:**
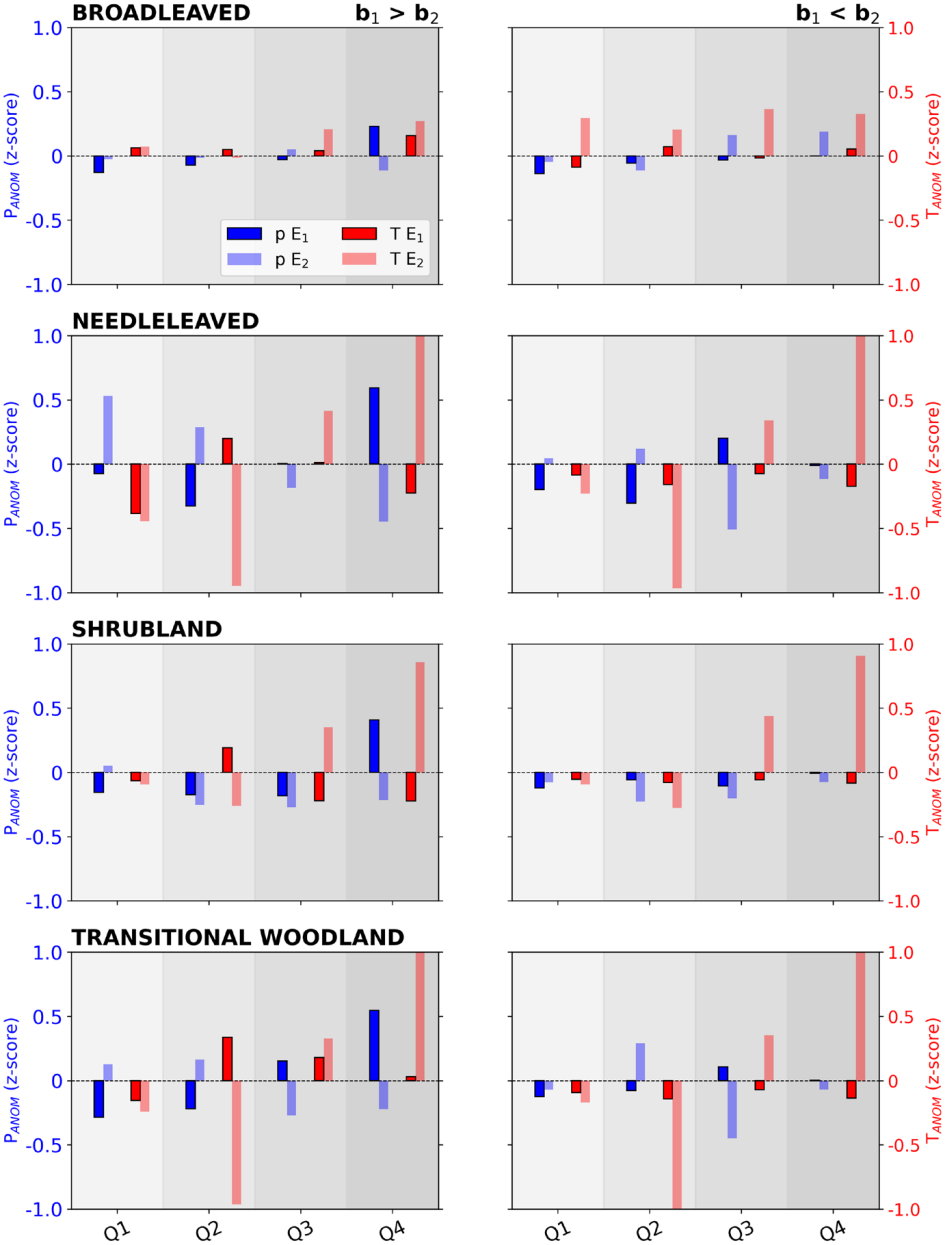
Post‐fire standardised anomalies (*z*‐score) of precipitation (blue bars and blue axis) and temperature (red bars and red axis) after E_1_ and E_2_. The anomalies are determined for both conditions, *b*_1_ > *b*_2_ (left panels) and *b*_1_ < *b*_2_ (right panels) in each land‐cover category, and for the following four quarters of months (Q1–Q4) after the fire occurrence. The shaded areas differentiate each quarter. The bars in bold are representative of the fire event whose pixels had a higher recovery rate.

Finally, climate variability during the recovery period might contribute to modulate recovery rates. We find that faster recovery of burned vegetation can be enhanced due to the availability of water or favourable temperatures, or even the conjunction of both conditions. By contrast, slow‐recovery pixels tend to be associated with negative anomalies of precipitation and high temperatures, with variations between vegetation types (Figure 6). For *Shb*, slow‐recovery rates are associated with precipitation deficits in the second quartile following the fire season (Q2, typically corresponding to local spring), in conjunction with positive temperature anomalies in the following Q3 (local summer) for all events analysed. The same is found for *BL* (*b*_1_ < *b*_2_). For the *BL*, *NL*, *Shb*, and *TW* pixels showing slower recovery rates following E_2_ than E_1_, the delay is predominantly associated with low rainfall with high temperatures in the autumn at the end of the subsequent growing season (Q4). By contrast, higher precipitation in Q4 results in faster recovery rates for *NL* and *Shb* following E_2_, compared to E_1_.

The persistence of positive precipitation anomalies immediately following the fire (Q1) can, however, promote a slower recovery rate, as seen in *NL* (*b*_1_ > *b*_2_).

## 5 Discussion

Here we provide a first assessment of vegetation recovery dynamics across the Mediterranean biome following repeated fires. We find predominantly faster recovery rates for broad‐leaved forests, compared to needle‐leaved forests, transitional woodlands and shrublands across the study region (Figure 1). Large variability in differences in recovery rates between the first and second fire events is found, explained by regional differences in vegetation type, but also post‐fire climate variability and fire severity. In general, mean recovery rate tends to be faster after E_1_ than E_2_, although regional differences are detected. Specifically, needle‐leaved forests of the western Mediterranean basin and southern California show predominantly faster recovery after E_2_ than E_1_. Burned vegetation from Australia and the Mediterranean basin, particularly broad‐leaved forests, is associated with higher recovery rates and no significant differences between fire events were detected. Broad‐leaved forests in the Mediterranean regions tend to be well adapted to fire, for example through resprouting ability or bark protection mechanisms, which allow for quick recovery of greenness following the fire (Pausas and Keeley 2009; Losso et al. 2022). Vegetation from South Africa, northern California and some areas of the eastern Mediterranean basin tend to be associated with slower recovery rates following E_2_ than E_1_, particularly due to the higher distribution of shrublands. As broad‐leaved forests, Mediterranean shrublands are also well adapted to fire, especially the ecosystems highly covered with obligate resprouters species. However, repeated fires can deplete resprouters species in ecosystems, which can be gradually replaced by more flammable shrubland populations such as obligate seeders. Together with post‐fire hotter and drier conditions, the obligate seeders communities can decrease the recovery rate of vegetation after recurrent fires and reduce the resilience of the ecosystem (Pausas and Keeley 2009; Keeley et al. 2012; Calvo et al. 2011; Salesa et al. 2022).

We find an emerging relationship between post‐fire recovery rates and relative fire severity across different vegetation types, with increasing dependence of recovery rates on fire severity with increasing fire severity (Figure 3). Mediterranean ecosystems tend to be adapted to fire, and some species have reproduction mechanisms that are activated by fire, such as the release and dispersal of seeds in soils from seed banks (Bright et al. 2019; Harrison et al. 2021; Landesmann et al. 2021; Falk et al. 2022). For similar levels of fire severity, and especially for extreme severity levels, most pixels tend to show slower recovery rates. Extremely severe fires, e.g., stand‐replacing fires, can eliminate or impair vegetation mechanisms to recover (Falk et al. 2022), and lead to low rates of burned ecosystems recovery. In this analysis, we have focused on pixels where repeated fires did not result in changes in vegetation type, which likely explains the characteristic shape of the bivariate distribution between fire severity and recovery rates.

We also find a modulation effect on post‐fire recovery rates by the pre‐fire vegetation conditions, through two pathways: (i) higher levels of pre‐fire greenness tend to be associated with highly severe fires, resulting in slower recovery rates; (ii) low levels of pre‐fire greenness tend to be associated with faster recovery rates. One plausible explanation for (i) is that high pre‐fire greenness, likely indicating healthy and productive vegetation, may be associated with dense live vegetation and significant fuel accumulation, available for combustion (Roberts et al. 2003; Parks et al. 2014). Thus, the fire propagation and severity can be exacerbated under summer hot and dry conditions, as pinpointed in several studies carried out on burned patches mainly composed of pines and eucalyptus in Mediterranean ecosystems (Gouveia et al. 2012, 2016; David et al. 2016; García‐Llamas et al. 2019; Ermitão et al. 2022), consequently leading to longer recovery periods. By contrast, larger departures from the ideal seasonal cycle prior to the fire event (ii) reflect loss of vegetation vitality or density prior to the fire event. In that case, faster recovery might be explained by reduced competition for resources between surviving or rapidly reestablishing vegetation. This might explain why the effect of pre‐fire condition is most pronounced in broad‐leaved forests, where competition for light following fire is likely to play an important role (Pausas and Keeley 2009).

We further showed that post‐fire climate conditions can modulate recovery rates, explaining differences in recovery between the first and second fires. Post‐fire water availability played a particularly important role, not only during the growing season following the fire event but also afterwards. A faster recovery was found for pixels with sufficient water availability and normal to slightly higher than climatological mean temperatures. These results are consistent with the fact that vegetation dynamics in these semi‐arid regions tends to be water‐limited (Gouveia, Trigo, and DaCamara 2009; Gouveia et al. 2017; Vicente‐Serrano 2006; Barkaoui et al. 2017; Ermitão et al. 2021; Rodrigues et al. 2024). For example, Meng et al. (2015) observed that the wet season after fire and temperatures in winter might be favourable in mixed conifer forests in California whereas Blanco‐Rodríguez et al. (2023) highlighted that post‐fire vegetation recovery in the first year is strongly conditioned by drought duration across western Mediterranean basin. We also show that rainfall deficits combined with high temperatures in the 9–12 months following the fire hampered recovery in all vegetation types. While increased water availability tends to favour recovery, we also found that persistent positive precipitation anomalies during the 3 months immediately after the fire were associated with slower recovery rates, especially for needle‐leaved forests. A possible explanation could be that excessive rainfall in the first months after the fire can increase top‐soil erosion and drag or destroy the seed banks released from burned trees, hampering the recovery capacity, as seen in Auld et al. (2020) after the 2019/2020 fire season in Australia. Post‐fire erosion events have been previously reported in several other regions, especially those with high slopes, such as central Portugal (Melo, van Asch, and Zêzere 2018), southern Italy (Esposito et al. 2023), or southern California (Cheung and Giardino 2023).

Our study shows that vegetation recovery is a complex process, depending on a large set of variables that are themselves interrelated, such as fire severity, pre‐fire vegetation condition and post‐fire climate variability. To better understand these relationships, long time‐series and finely resolved information in space is needed. We analysed the areas that burned twice, as the fire return periods for the Mediterranean regions are on average 20 years (Archibald et al. 2013; Harrison et al. 2021). Hence, this constrains our approach because our time‐series are still quite short (about 20 years) to assess recovery in other regions of the world where fire return intervals and recovery are much longer (50–100 years), such as the boreal regions (Harrison et al. 2021). Furthermore, our approach for recovery estimation is based on the vegetation greenness index EVI, which effectively captures the impact of the fire on vegetation, as it is highly sensitive to fire events and reflects the initial stages of vegetation recovery through leaf resprouting (Bousquet et al. 2022). However, we note that EVI cannot fully capture the restoration of aboveground biomass (AGB) and carbon (AGC), particularly since AGB, which is heavily impacted by fire, often begins to recover only several months after the event, as seen in Bousquet et al. (2022) among different biomes and in Fan et al. (2023) over boreal regions. This limitation could be overcome by using other satellite‐based datasets that better capture aboveground biomass dynamics such as Radar, or LiDAR, but such datasets are only available at very coarse spatial resolution and have shorter temporal coverage (Pérez‐Cabello, Montorio, and Alves 2021; Qin et al. 2022; Fan et al. 2023), that is not compatible with the aim of long term recovery and resilience assessment.

We note that other fire‐related variables, such as topography, biomass accumulation, land management, or human intervention, are also likely to affect the recovery of burned ecosystems, and can be considered in future studies. On the other hand, despite the quality analysis, we are aware that land‐cover products may have classification errors that can not fully capture changes in vegetation, constituting a source of uncertainty, although we use an approach that aims to ensure that the land cover‐type is the same before both fire events, partly mitigating the effect of land‐cover shifts in the specific pixel. Moreover, it should also be stressed that we are interested in evaluating the recovery rate of greenness considering different vegetation types frequently disturbed by wildfires within Mediterranean ecosystems, and we do not work towards the estimation of specific recovery times and their phenology. Notwithstanding, our approach shows the value of estimating recovery dynamics based on time‐series rather than space‐for‐time substitution, analysing how pre‐fire conditions and fire severity modulate recovery dynamics comparing recurrent events. More frequent or intense hot and dry conditions under climate change can hamper the recovery process, especially in regions with typically high fuel loads, which can in turn result in severe and prolonged damage to ecosystems. While Mediterranean ecosystems are adapted to fire, changes in fire recurrence and increasing water deficits can further limit vegetation's ability to recover, by reducing, for example, post‐fire seedling densities (Rodman et al. 2020).

## 6 Conclusions

This study proposes an assessment of how the Mediterranean biome throughout the world has been recovering from recurrent fires, relying on a mono‐parametric statistical model applied to a time‐series of spectral index EVI that evaluates the rate of recovery of vegetation greenness following fires. Although well adapted to fire, the Mediterranean ecosystems under climate change are vulnerable to increasing fire severity and unfavourable post‐fire conditions, making it essential to understand the multiple factors that influence vegetation recovery dynamics.

Here, we find that the mean recovery rate of burned vegetation tends to be faster after the first event than the second event, although we detect large variability between recovery rates, which are roughly attributed to regional contrasts in different vegetation types, and also fire severity and post‐fire climate conditions. In particular, needle‐leaved forests tend to recover faster after the second event, whereas shrublands contrast by showing faster recovery following the first event. Broad‐leaved forests and transitional woodlands show no statistically significant differences in recovery rate distributions, although their broad spread suggests marked regional differences, particularly in areas such as in the Mediterranean basin.

Our results show that fire severity modulates recovery dynamics, as recovery rates are enhanced with increasing severity. However, extremely severe fires are followed by very slow recovery, and these events are strongly associated with high values of pre‐fire greenness of vegetation, particularly in forest ecosystems. We further find that post‐fire climate variability modulates the rates of vegetation recovery. Precipitation availability, associated with normal to above‐mean temperatures in the growing season, seems to favour vegetation greenness recovery. This reflects the importance of water availability in these ecosystems and shows how precipitation and temperature changes in the future might affect the regeneration of recurrently burned ecosystems.

The overall consistency of the results shows the good performance of the proposed methodology, which enhances the value of using a time‐series approach combined with a mono‐parametric model to determine the recovery rates of burned vegetation, rather than space‐for‐time substitution methods, providing a reliable tool for understanding post‐fire recovery dynamics.

## Author Contributions

**Tiago Ermitão:** conceptualization, data curation, formal analysis, investigation, methodology, writing – original draft, writing – review and editing. **Célia M. Gouveia:** conceptualization, formal analysis, investigation, methodology, resources, writing – review and editing. **Ana Bastos:** conceptualization, formal analysis, investigation, writing – review and editing. **Ana C. Russo:** conceptualization, funding acquisition, investigation, project administration, resources, writing – review and editing.

## Conflicts of Interest

The authors declare no conflicts of interest.

## Supporting information


**Figure S1.** Monthly distribution of precipitation in mm month‐1 (dark blue bars) and monthly temperature in °C (red line) for each of the regions within the domain. The numbered labels are: 1—Mediterranean basin; 2—California; 3—Australia; 4—South Africa; 5—Chile.

**Figure S2.** Boxplots of the distribution of the *r* ^2^ used to evaluate the model’s performance for each land cover, considering the first fire event (green) and the second fire event (brown).

**Figure S3.** Maps of the distribution of *r* ^2^ used to evaluate the model performance in each of regions of the study. The numbered labels are: 1—Mediterranean basin; 2—California; 3—Australia; 4—South Africa; 5—Chile.

**Figure S4.** Kernel density estimates (KDEs) of the bivariate distribution of recovery rate (*b*) and the correspondent confidence intervals (CI) at level‐confidence of 95%. The KDEs are plotted for the four land‐cover categories, considering the first, E_1_, and the second, E_2_, events.

**Figure S5.** Spatial distribution of the fire frequency in each of the regions within the study area. The pixels which burned five or more times over the past 22 years are represented in black. The numbered labels are: 1—Mediterranean basin; 2—California; 3—Australia; 4—South Africa; 5—Chile.

**Figure S6.** Maps of the recovery rate (*b*) of vegetation over the pixels that burned twice within the period 2001–2022 following the first fire event in each of the regions of the domain. The numbered labels are: 1—Mediterranean basin; 2—California; 3—Australia; 4—South Africa; 5—Chile.

**Figure S7.** Same as Figure S6, but following the second fire event. The numbered labels are: 1—Mediterranean basin; 2—California; 3—Australia; 4—South Africa; and 5—Chile.

**Figure S8.** Distribution of differences in recovery rates between the two fire events, b_DIFF_ for broad‐leaved forests (BL) and transitional woodlands (TW) of Mediterranean basin (left panels), Australia (middle panels), and South Africa (right panels). The black dashed line describes the median value of b_DIFF_ and the thin grey dashed line is the vertical line of *b*_DIFF_ = 0. The *p*‐value corresponds to the Wilcoxon signed‐rank test applied with a 95% significance.

## Data Availability

The data and code used to generate the main results of this study are available in Zenodo at https://doi.org/10.5281/zenodo.14477670. EVI and burn area data were obtained from the NASA EOSDIS Land Processes Distributed Active Archive Center at https://doi.org/10.5067/MODIS/MOD13A1.061 and https://doi.org/10.5067/MODIS/MCD64A1.061, respectively. Land cover data were obtained from the ESA Climate Change Initiative and in particular its Land Cover project at https://maps.elie.ucl.ac.be/CCI/viewer/download.php (version 2.0.8). Precipitation and air temperature were obtained from Copernicus at https://doi.org/10.24381/cds.e2161bac.
