# Supplementary figures and images for: Recovery Following Recurrent Fires Across Mediterranean Ecosystems
Source: Glob Chang Biol. 2024 Dec 27;30(12):e70013. doi: 10.1111/gcb.70013 (PMC11671852; doi:10.1111/gcb.70013)

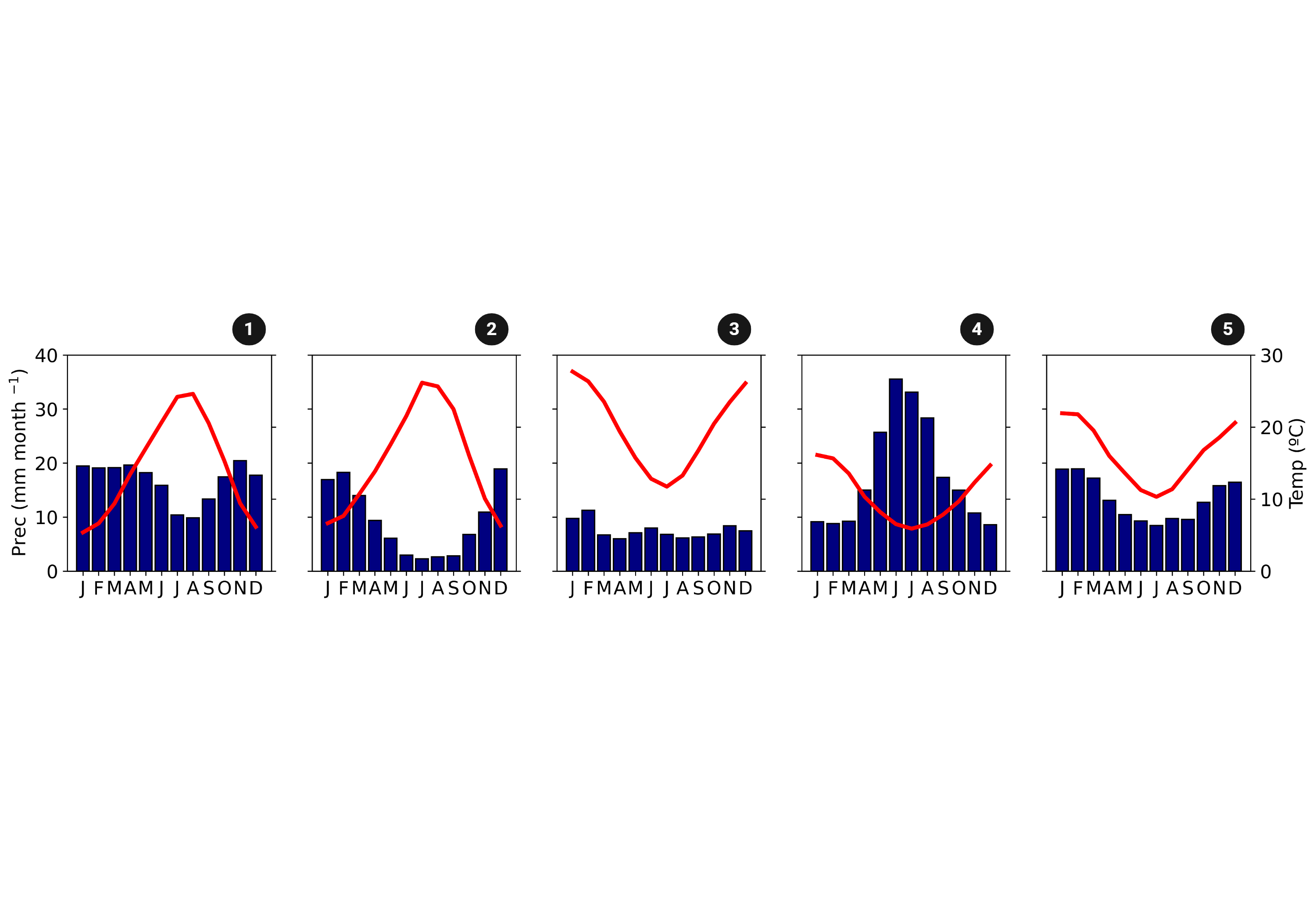

Supplement: Supplementary file 1 — Figure S1. Monthly distribution of precipitation in mm month‐1 (dark blue bars) and monthly temperature in °C (red line) for each of the regions within the domain. The numbered labels are: 1—Mediterranean basin; 2—California; 3—Australia; 4—South Africa; 5—Chile. [file GCB-30-e70013-s008.png]

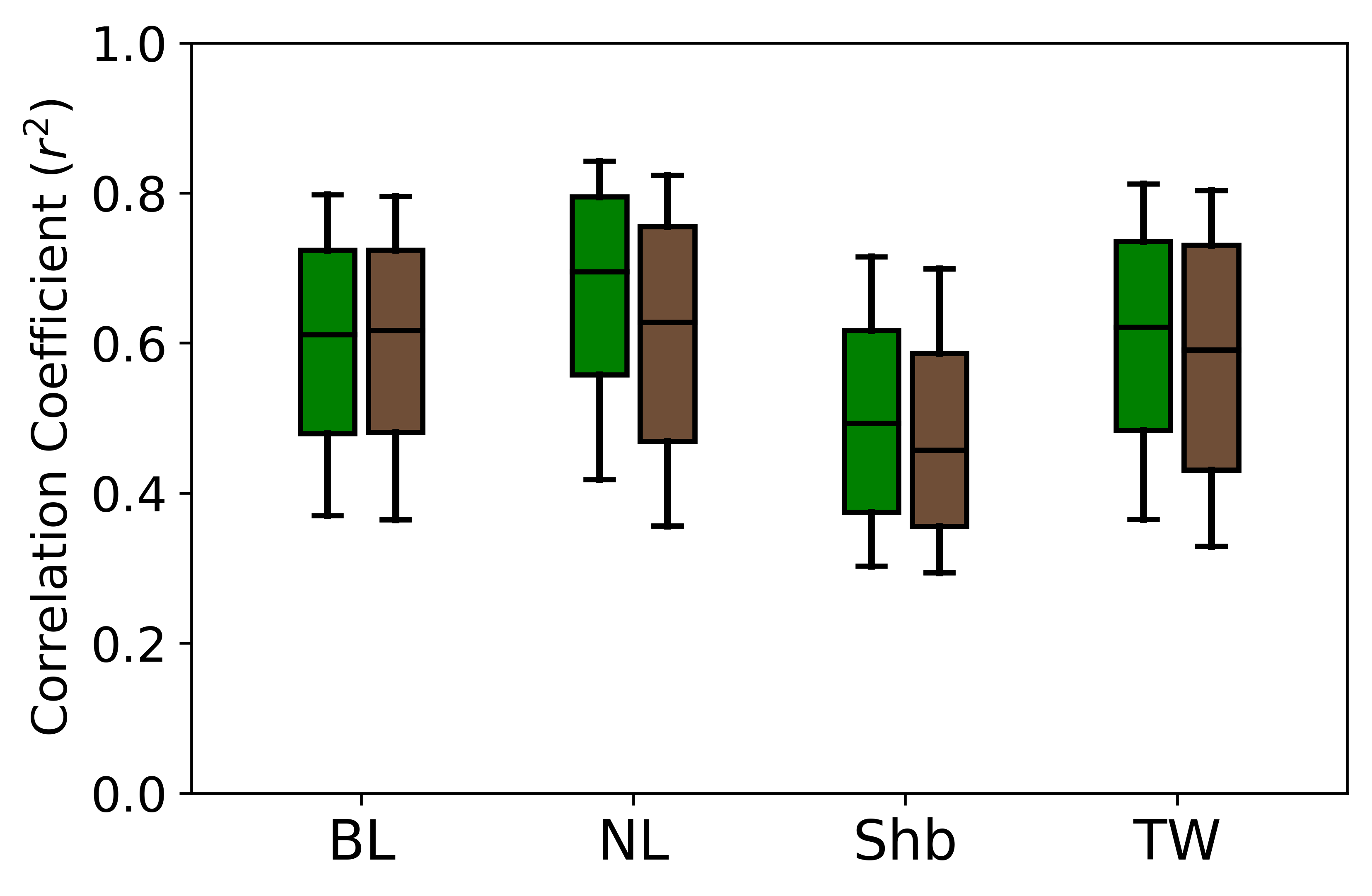

Supplement: Supplementary file 2 — Figure S2. Boxplots of the distribution of the r 2 used to evaluate the model’s performance for each land cover, considering the first fire event (green) and the second fire event (brown). [file GCB-30-e70013-s003.png]

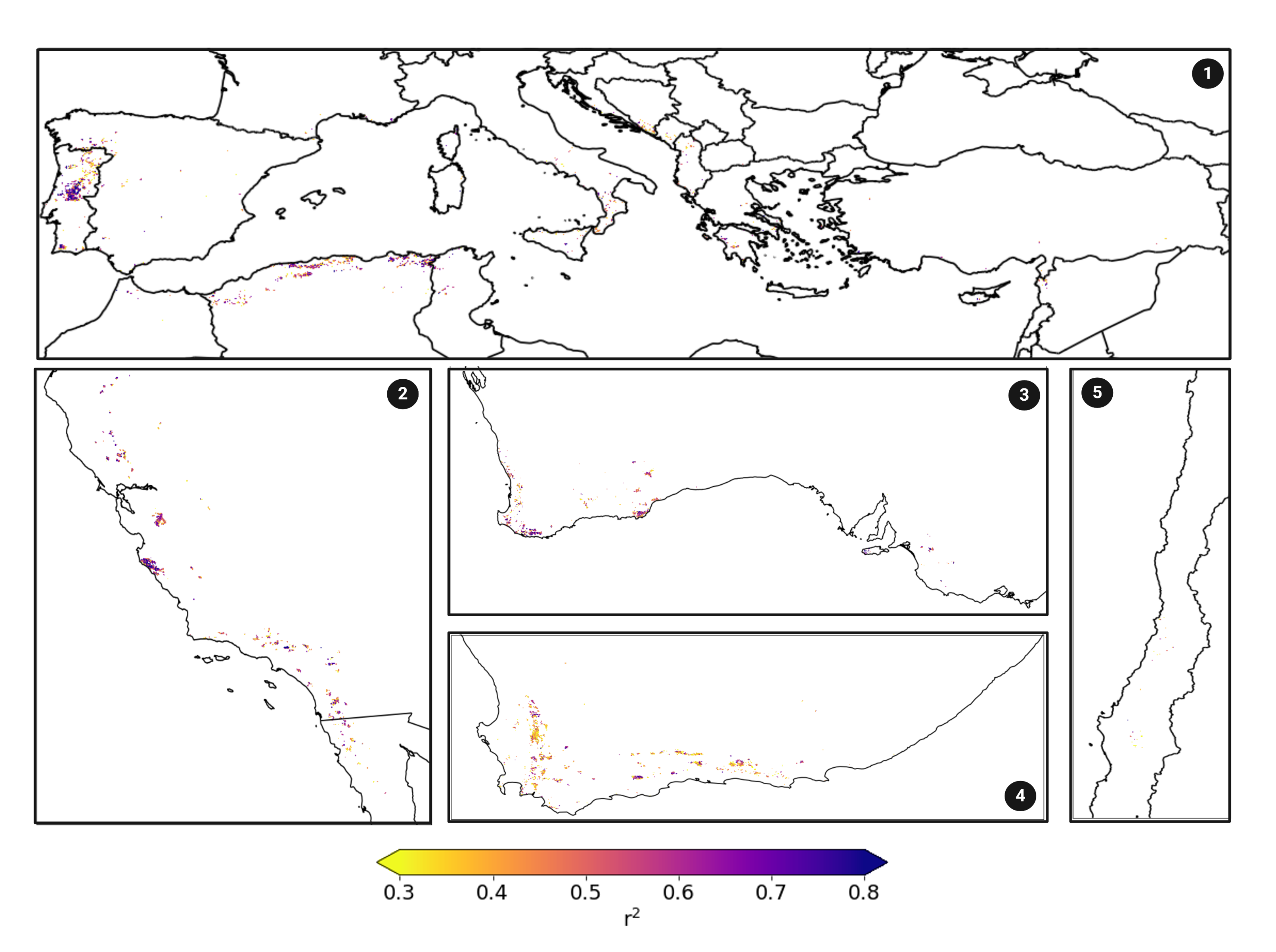

Supplement: Supplementary file 3 — Figure S3. Maps of the distribution of r 2 used to evaluate the model performance in each of regions of the study. The numbered labels are: 1—Mediterranean basin; 2—California; 3—Australia; 4—South Africa; 5—Chile. [file GCB-30-e70013-s007.png]

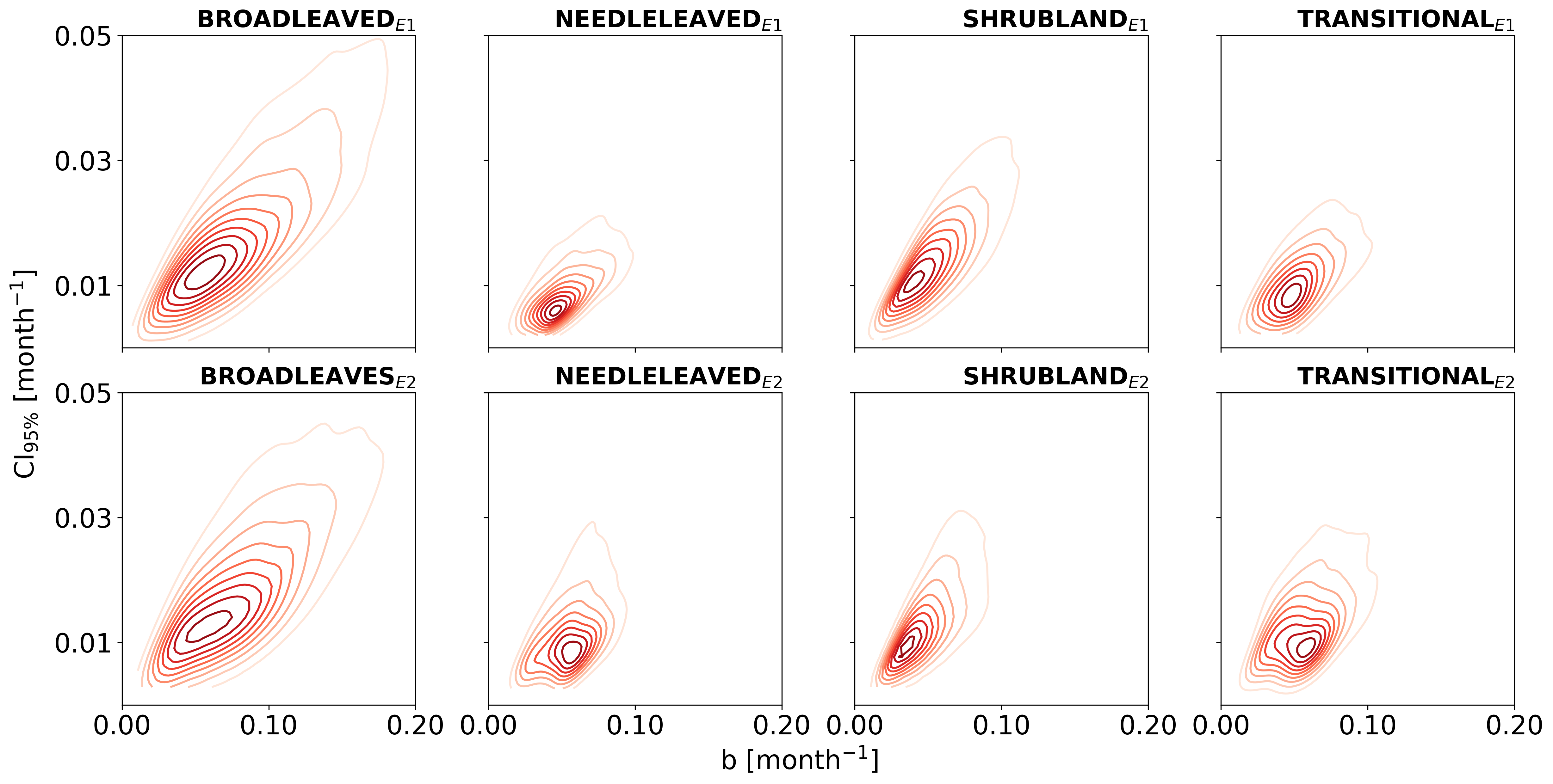

Supplement: Supplementary file 4 — Figure S4. Kernel density estimates (KDEs) of the bivariate distribution of recovery rate (b) and the correspondent confidence intervals (CI) at level‐confidence of 95%. The KDEs are plotted for the four land‐cover categories, considering the first, E1, and the second, E2, events. [file GCB-30-e70013-s006.png]

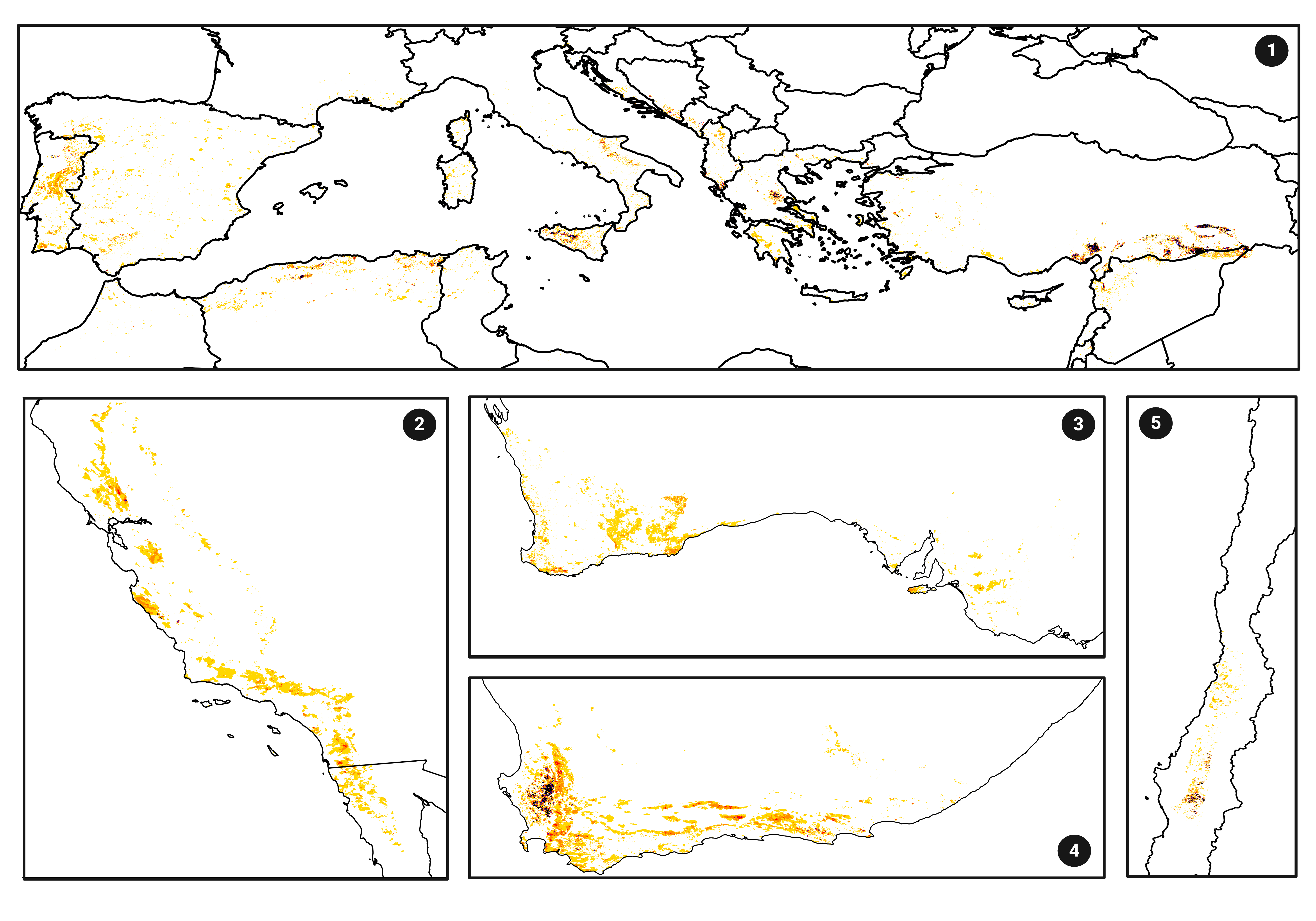

Supplement: Supplementary file 5 — Figure S5. Spatial distribution of the fire frequency in each of the regions within the study area. The pixels which burned five or more times over the past 22 years are represented in black. The numbered labels are: 1—Mediterranean basin; 2—California; 3—Australia; 4—South Africa; 5—Chile. [file GCB-30-e70013-s001.png]

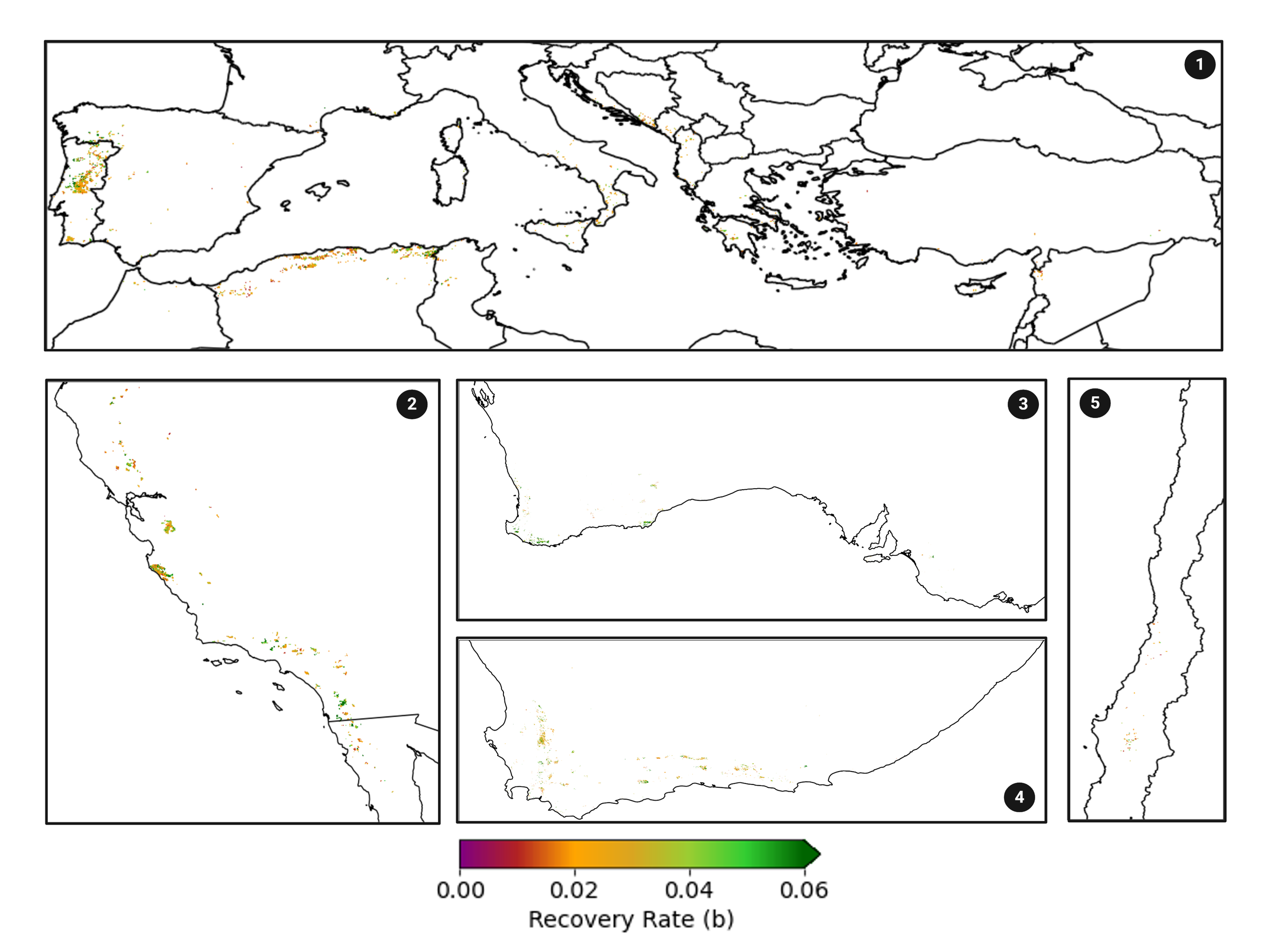

Supplement: Supplementary file 6 — Figure S6. Maps of the recovery rate (b) of vegetation over the pixels that burned twice within the period 2001–2022 following the first fire event in each of the regions of the domain. The numbered labels are: 1—Mediterranean basin; 2—California; 3—Australia; 4—South Africa; 5—Chile. [file GCB-30-e70013-s002.png]

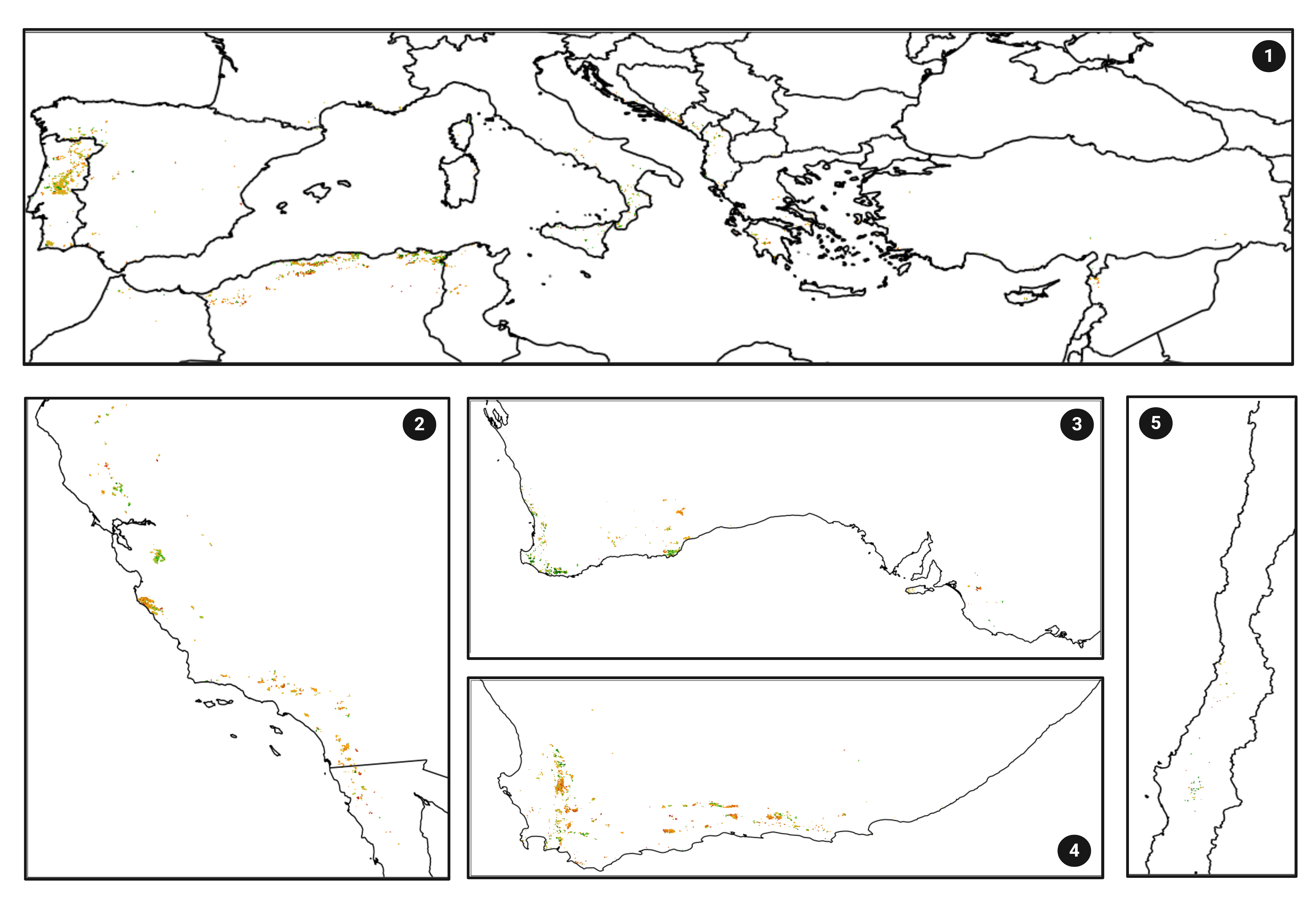

Supplement: Supplementary file 7 — Figure S7. Same as Figure S6, but following the second fire event. The numbered labels are: 1—Mediterranean basin; 2—California; 3—Australia; 4—South Africa; and 5—Chile. [file GCB-30-e70013-s004.png]

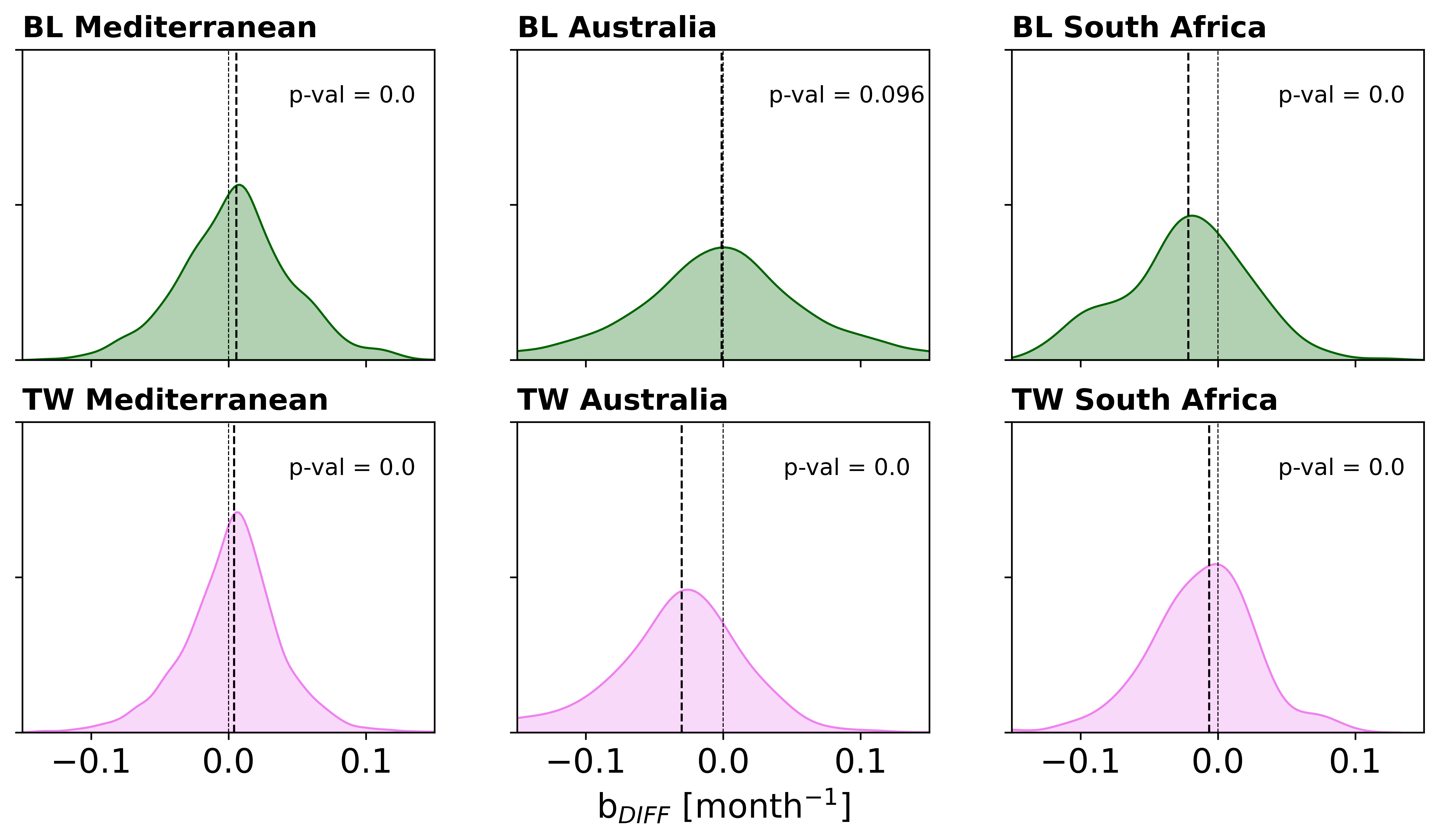

Supplement: Supplementary file 8 — Figure S8. Distribution of differences in recovery rates between the two fire events, bDIFF for broad‐leaved forests (BL) and transitional woodlands (TW) of Mediterranean basin (left panels), Australia (middle panels), and South Africa (right panels). The black dashed line describes the median value of bDIFF and the thin grey dashed line is the vertical line of b DIFF = 0. The p‐value corresponds to the Wilcoxon signed‐rank test applied with a 95% significance. [file GCB-30-e70013-s005.png]
